# SPACA9 is a lumenal protein of human ciliary singlet and doublet microtubules

**DOI:** 10.1073/pnas.2207605119

**Published:** 2022-10-03

**Authors:** Miao Gui, Jacob T. Croft, Davide Zabeo, Vajradhar Acharya, Justin M. Kollman, Thomas Burgoyne, Johanna L. Höög, Alan Brown

**Affiliations:** ^a^Department of Biological Chemistry and Molecular Pharmacology, Harvard Medical School, Boston, MA 02115;; ^b^Department of Chemistry and Molecular Biology, University of Gothenburg, Gothenburg 41390, Sweden;; ^c^Department of Biochemistry, University of Washington, Seattle, WA 98195;; ^d^Royal Brompton Hospital, Guy's and St Thomas' NHS Foundation Trust, London SW3 6NP, United Kingdom;; ^e^Institute of Ophthalmology, University College London, London EC1V 9EL, United Kingdom

**Keywords:** cilia, microtubules, axoneme, cryo-EM, cryo-ET

## Abstract

The cilium-centrosome complex contains triplet, doublet, and singlet microtubules. The lumenal surfaces of each microtubule within this diverse array are decorated by microtubule inner proteins (MIPs). Here, we used single-particle cryo-electron microscopy methods to build atomic models of two types of human ciliary microtubule: the doublet microtubules of multiciliated respiratory cells and the distal singlet microtubules of monoflagellated human spermatozoa. We discover that SPACA9 is a polyspecific MIP capable of binding both microtubule types. SPACA9 forms intralumenal striations in the B tubule of respiratory doublet microtubules and noncontinuous spirals in sperm singlet microtubules. By acquiring new and reanalyzing previous cryo-electron tomography data, we show that SPACA9-like intralumenal striations are common features of different microtubule types in animal cilia. Our structures provide detailed references to help rationalize ciliopathy-causing mutations and position cryo-EM as a tool for the analysis of samples obtained directly from ciliopathy patients.

## Significance

Cilia are cell-surface organelles with cytoskeletons formed by different microtubule types. These microtubules are decorated inside and out by proteins that alter microtubule stability and elasticity and allow cilia to beat. Mutations in these proteins are associated with human ciliopathies such as primary ciliary dyskinesia. Here, we used cryo-EM to reveal the structures of two distinct types of human ciliary microtubule: the doublet microtubules of respiratory tract cilia and the distal singlet microtubules of the sperm tail. Among the microtubule-binding proteins identified is SPACA9, which we show is capable of forming both spirals and striations within human ciliary microtubules. The ability to resolve human ciliary microtubule composition improves our understanding of ciliary complexes and the potential causes of human ciliopathies.

Cilia contain complex assemblies of different microtubules. These microtubules are essential for the organization and motility of the cilium and provide tracks for intraciliary protein transport. At the base of a cilium is the basal body, a modified centriole consisting of a circular arrangement of nine triplet microtubules (TMTs). The A and B tubules of TMTs transition into the doublet microtubules (DMTs) of the axoneme. In motile cilia, these axonemal DMTs anchor thousands of dynein motors and regulatory complexes that together generate ciliary motility. DMTs also often surround a central apparatus (CA), an additional structure of two heavily patterned and interconnected microtubules, C1 and C2. DMTs themselves often transition into singlet microtubules (SMTs) near the ciliary tip, with the length of this singlet zone varying between species and cell types (1). For example, the singlet zone in immotile (primary) cilia often exceeds the length of the DMTs from which they originate (2, 3), whereas the motile flagellum of *Trypanosoma brucei* does not contain a singlet zone at all (4, 5). In cilia that do have a singlet zone, the transition from DMT to SMT either involves the abrupt termination or selective loss of the distal region of the B tubule or the splitting of A and B tubules into 2 separate microtubules, each with 13 protofilaments (6). B tubule loss seems to predominate in mammalian primary cilia (2, 3), whereas splitting has been observed in the motile flagella of rodent and human spermatozoa (7–9).

The lumenal surfaces of the TMTs, DMTs, and SMTs of motile cilia are patterned by microtubule inner proteins (MIPs). The identities of some of these MIPs have become known through cryo-electron microscopy (cryo-EM) studies of DMTs from the model organism *Chlamydomonas reinhardtii* (10). We recently revealed that many of these MIPs are conserved in the DMTs of bovine respiratory cilia (11). In addition to near-universal MIPs, a subset of MIPs is specific to mammals. A possible function of MIPs is to fine-tune the physical properties of microtubules—for example, to modulate stability or elasticity (12). Because the required properties of ciliary microtubules may differ depending on the morphology of cilia, their function, and the environment in which they operate, this raises the question of whether different cilium types within an organism have different MIP repertoires. In humans, for example, the motile cilia of tracheal epithelia are an order of magnitude shorter than the motile flagella of spermatozoa (6 μm compared with 60 μm) (13, 14). The cilia of these two cell types also have different functions: respiratory cilia beat in metachronal waves to expel inhaled pathogens and particles, whereas sperm flagella evolved to propel spermatozoa through the female reproductive tract. Cell-specific specialization of axonemal structure within an organism is supported by the recent discovery that human sperm and airway cells have distinct compositions of axonemal dynein (15). Differences in the composition of axonemes between cell types may explain the tissue-specific effects on ciliary motility of mutations in some axonemal components (reviewed by Sironen et al. (16)). Resolving ciliary microtubule composition therefore has important implications for our ability to understand the phenotypic manifestation of human ciliopathies, which are associated with varying levels of infertility, respiratory disease, left–right body asymmetry, and heterotaxy (17).

Compared with DMTs, the identities of MIPs in other ciliary microtubules are less well understood. Recent structures of *C. reinhardtii* CA microtubules (18, 19) have shown that they have a mutually exclusive set compared with DMTs. It may be expected that the MIPs of the TMTs and SMTs are identical to those of the DMTs with which they are contiguous. However, cryo-electron tomography (cryo-ET) studies of mammalian axonemes indicate that these microtubules have different patterns of MIPs (6, 20). We have previously used cryo-ET to show that the lumens of SMTs of human spermatozoa contain a structure called TAILS (tail axoneme intralumenal spiral) (6). This structure forms a noncontinuous left-handed spiral with an 8-nm pitch that breaks at the microtubule seam. TAILS forms not only in SMTs but up to 300 nm into the distal DMT (6). However, the resolution provided by cryo-ET was insufficient to identify the component or components responsible for TAILS.

To address the question of how MIP repertoires differ among ciliary microtubules within an organism, we have used single-particle cryo-EM to determine the structures of human ciliary microtubules from two different cell types: the SMTs of spermatozoa and the DMTs of ciliated epithelial cells of the respiratory tract. Comparison of the structures of human and bovine respiratory DMTs (11) identified six additional MIPs, including SPACA9, which forms striations in the B tubule of human airway cilia. A cryo-EM structure of human sperm SMTs demonstrated that SPACA9 is also responsible for TAILS. Striations resembling those formed by SPACA9 are visible in tomographic reconstructions of other microtubules, leading us to speculate that SPACA9 could be a promiscuous binder of mammalian ciliary microtubules.

## Results

### The MIP Repertoire of Human Respiratory DMTs.

**Fig. 1.**
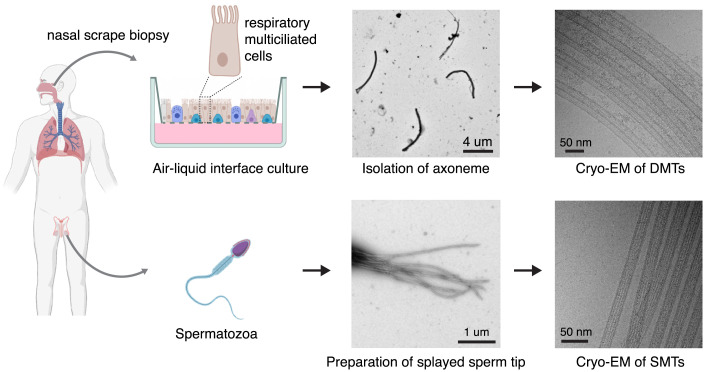
Sources of human ciliary microtubules for cryo-EM analysis. Human ciliary microtubules were studied from two sources: multiciliated respiratory cells grown at the ALI and differentiated from basal cells obtained by nasal scrape biopsy (*Top*), and from the distal tips of spermatozoa (*Bottom*). Analysis of DMTs from respiratory cilia required isolating axonemes from the ALI cultures. The SMTs of sperm axonemes were imaged directly from demembranated sperm cells. Schematics were created with BioRender.

To determine the MIP composition of human DMTs, we collected airway epithelial cells from a healthy volunteer by noninvasive nasal scrape biopsy (Fig. 1). The cells were subsequently redifferentiated into pseudostratified mucociliary epithelia by culturing them at the air–liquid interface (ALI) for 28 d. The cilia were stripped from the ALI cultures (21) and their axonemes recovered for vitrification onto cryo-EM grids. The application of axonemes onto the cryo-EM grid and blotting during the freezing process splayed the axonemes into individually resolved, and therefore imageable, DMTs (Fig. 1, *Top Right*). In total, we collected 16,933 useable micrographs, from which we determined a 3.6-Å resolution structure of the 48-nm internal repeat of the DMT (*SI Appendix*, Fig. S1 and S2*A* and Table S1). Focused refinements improved the resolution further so that all protofilaments are resolved to between 3.1 and 3.5 Å (*SI Appendix*, Fig. S2 *A* and *B*). At this resolution, side chains are resolved (*SI Appendix*, Fig. S2 *C–E*) and accurate atomic models can be built.

**Fig. 2.**
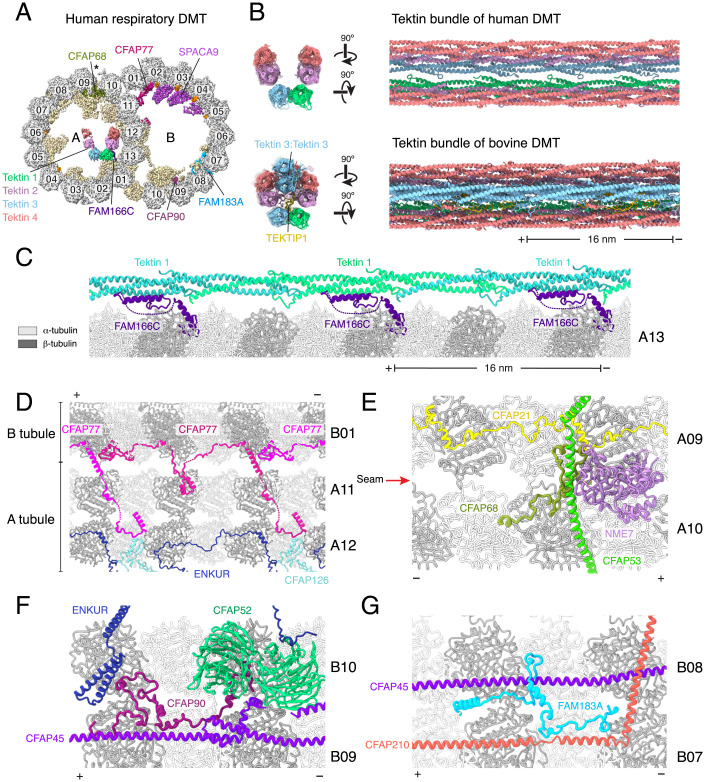
Structure of the human respiratory DMT identifies MIPs. (*A*) Cross-section of the cryo-EM density map of the 48-nm repeat of the human respiratory DMT. MIPs previously identified in the bovine respiratory DMT (11) are pale yellow. Tektins and newly identified MIPs are shown with unique colors and labeled. Unidentified SAXO proteins are shown in orange. Protofilaments are shown in gray and numbered according to convention. The seam of the A tubule is marked with an asterisk. (*B*) Two views comparing the tektin bundles of human (*Top*) and bovine (*Bottom*) respiratory DMTs. Compared to the bovine structure, the human tektin bundle lacks a tektin 3 homodimer and TEKTIP1. (*C*) FAM166C binds tektin 1 and may play a role in positioning the tektin bundle within the A tubule. (*D*) CFAP77 links the A and B tubules from within the B tubule. On the A tubule, CFAP77 forms a small interface with CFAP126, a previously identified MIP. (*E*) CFAP68 binds to the seam of the A tubule, where it contacts a number of other MIPs, including CFAP53, CFAP68, and NME7. (*F*) CFAP90 binds within a cluster of MIPs near the inner junction. These proteins include CFAP45, CFAP52, and ENKUR. (*G*) FAM183A connects the filamentous MIPs CFAP45 and CFAP210 on protofilaments B07 and B08 of the B tubule. In panels (*B*)*–*(*G*), plus and minus signs indicate the polarity of the microtubule.

The structure revealed that the MIP repertoire of human DMTs is similar to that of native bovine trachea cilia (11). We take this similarity to indicate that not only are MIPs conserved between mammals but also that cilia grown in ALI cultures recapitulate the features of native cilia collected directly from nasal tissue. In total, we unambiguously identified 33 MIPs (*SI Appendix*, Table S2). Many of these MIPs were identified in identical positions in the bovine DMT structure (11) and will not be discussed further here. However, we also identified six additional MIPs: C11orf1, CFAP77, C5orf49, FAM166C, FAM183A, and SPACA9 (Fig. 2*A*). These MIPs were identified by comparing de novo built polyalanine models with entries in the Protein Data Bank (PDB) or the AlphaFold2 Protein Structure Database (22) using Foldseek (23) or manually against a smaller AlphaFold2 structure library (*SI Appendix*, Fig. S3) generated from the mass spectrometry of purified human axonemes (Dataset S1). The solutions were verified based on the fit of side chains into the cryo-EM density map (*SI Appendix*, Fig. S2). C11orf1 and C5orf49 are structurally similar to the *C. reinhardtii* MIPs FAP68 and FAP90 and bind similar sites of the DMT (10), so we renamed these proteins CFAP68 and CFAP90, respectively, with approval from the HUGO Gene Nomenclature Committee. Orthologs of the genes encoding CFAP77 and FAM183A are also found in the *C. reinhardtii* genome, leading us to speculate that these proteins in *Chlamydomonas* (FAP77 and FAP144) could be DMT MIPs not resolved in our previous study (10).

Of the six additional mammalian MIPs, only SPACA9 is unique to human respiratory DMTs; the others are present in the bovine DMT map (EMD-24664) (11) but could not be identified in the prior study due to lower map quality. Despite the overall conservation of the MIP repertoire, the tektin-3 homodimer that forms the apex of a pentagonal arrangement of tektin filaments within the A tubule lumen of bovine DMTs is missing in the human DMT (Fig. 2 *A* and *B*). Tektin bundle interacting protein 1 (TEKTIP1) is also absent. This simplified arrangement of six rather than eight tektin filaments could be a true difference between human and bovine respiratory DMTs. This possibility is supported by data from the Human Protein Atlas showing that TEKTIP1 expression is predominantly restricted to sperm cells. Along with classes with 6 tektin filaments (20% of the data), we observed classes corresponding to no tektin (47%) and 3 to 4 tektin filaments (33%). These distinct classes may reflect heterogeneity within the cilia population, but we cannot rule out these simplified tektin architectures being artifacts of a sample-preparation process that involves both detergent treatment and mechanical force that may disrupt the polar interactions between tektin filaments.

Of these 6 additionally identified MIPs, FAM166C may play a role in ensuring the correct placement of the tektin bundle, as it has the same 16-nm periodicity as tektin and an α helix that pairs with tektin-1 (Fig. 2*C*). CFAP77 forms a connection between the A and B tubules on the inside of the outer junction (Fig. 2*D*), CFAP68 binds at the microtubule seam (Fig. 2*E*), and CFAP90 and FAM183A bind to adjacent protofilaments of the B tubule (Fig. 2 *F* and *G*).

### SPACA9 Forms B Tubule Striations.

**Fig. 3.**
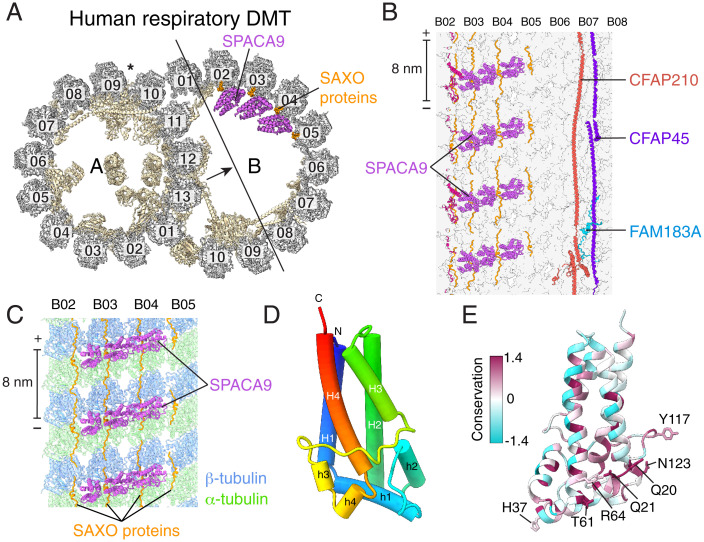
SPACA9 binds the B tubule of the respiratory doublet microtubule (DMT). (*A*) Cross section of the atomic model of the human respiratory DMT with tubulin colored gray, MIPs colored pale yellow, SPACA9 colored purple, and SAXO proteins colored orange. The arrow shows the direction of view in panel *B*. (*B*) Longitudinal section of the B-tubule atomic model viewed from the lumen showing striations of SPACA9 bound to protofilaments B02-B05 and other MIPs bound to protofilaments B06-B08. The polarity of the microtubule is shown at the ends of the scale bar. (*C*) SPACA9 binds at the intradimer interface between α- and β-tubulin and repeats with 8-nm longitudinal periodicity. (*D*) Atomic model of a single SPACA9 molecule with helices displayed as tubes and colored in rainbow from C- to N terminus. (*E*) Atomic model of SPACA9 colored by sequence conservation. Conserved residues are shown in magenta. Conserved residues that interact with tubulin and SAXO protein(s) are labeled.

SPACA9, the single unique component of human respiratory DMTs relative to bovine DMTs, binds in an arc of three proteins to protofilaments B02-B05 (Fig. 3*A*). This trimeric arc locates at the intradimer interface between α- and β-tubulin and repeats with 8-nm longitudinal periodicity, generating a highly distinctive striation pattern (Fig. 3 *B* and *C*). The density for SPACA9 between protofilaments B04 and B05 is less well resolved than the two preceding copies (*SI Appendix*, Fig. S2*B*), suggesting flexibility or substoichiometric binding. SPACA9 is likely restricted to at most protofilaments B02 to B06 because other MIPs, including CFAP210 and FAM183A, bind protofilaments B07 to B10 (Fig. 3*B*).

The identification of SPACA9 as a microtubule-binding protein explains its detection in a high-throughput immunofluorescence screen of proteins that colocalize with microtubules (24) and in the proteome of human airway cilia (25). SPACA9 has an unusual but simple fold consisting of eight α helices arranged as a 4-helix bundle (H1 to H4) with insertions after H1 and H3 containing 2 short helices apiece (Fig. 3*D*). These shorter helices (h1 to h4) cap the 4-helix bundle and generate the microtubule-binding surface. Searches for similar folds in the PDB or the AlphaFold2 Protein Structure Database (22) using Foldseek (23) were unsuccessful, indicating that SPACA9 has a unique fold among human proteins. Sequence conservation suggests that the fold is conserved in animals across the phylogenetic tree (*SI Appendix*, Fig. S4).

Because the striated densities generated by SPACA9 in the B tubule are distinctive and have an 8-nm periodicity, which is rare among mammalian DMT MIPs, we analyzed previously published subtomogram averages of DMTs from other organisms and cell types for similar densities (*SI Appendix*, Fig. S5). SPACA9-like densities were found in the B tubule of the human respiratory DMT (21), as expected, as well as in the subtomogram averages of B tubules of horse and pig sperm DMTs (26). In contrast, repetitive B tubule striations were not observed in the bovine respiratory DMT (20) or the mouse sperm DMT (26). These results indicate that SPACA9-like striations can occur in both respiratory and sperm DMTs, but they are not universal among animals. High-resolution structures will be needed to confirm the presence of SPACA9 in other animal DMTs.

**Fig. 4.**
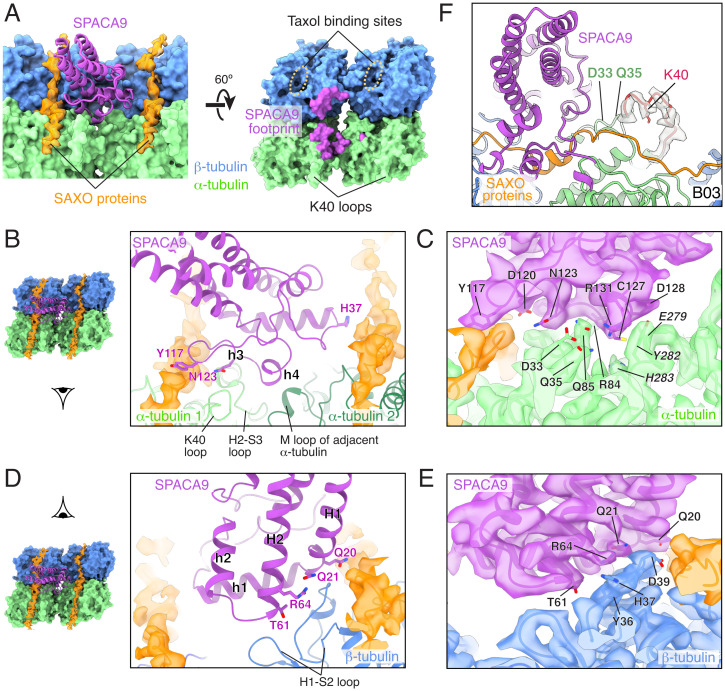
Interaction of SPACA9 and microtubule. (*A*, *Left*) model of SPACA9 (cartoon, purple) engaged with parallel SAXO proteins (map density, orange) and the intradimer and interprotofilament interfaces of α/β-tubulin (surface, blue and green, respectively). *Right*, the footprint of SPACA9 (purple) on a tubulin tetramer. (*B*) View of the conserved residues of SPACA9 that interact with two neighboring α-tubulin molecules (light and dark green) and the unidentified SAXO protein(s). (*C*) Density map and side chains showing the interface between SPACA9 and α-tubulins. (*D*) A view of the conserved residues of SPACA9 that interact with β-tubulin and the unidentified SAXO protein(s). The schematics to the left of panels (*B*) and (*D*) indicate the viewing angles of the enlarged views. (*E*) Density map and side chains showing the interface between SPACA9 and β-tubulin. (*F*) Transparent density map and fitted model showing that the αK40 loop of α-tubulin is ordered in the presence of SPACA9. The αK40 loop (residues 37 to 47) and the side chain of K40 are in red. The density map and model are from the B03 protofilament. Density maps, sharpened in Phenix (27), are shown in panels (*C*)*,* (*E*), and (*F*).

The footprint of SPACA9 on the human respiratory DMT involves both α- and β-tubulins of one protofilament and an α-tubulin of the adjacent protofilament with an interface area of ∼1,000 Å^2^ (Fig. 4*A*). The binding is maintained predominantly by polar interactions that involve several conserved residues of SPACA9, including Q20, Q21, T61, R64, and N123 (Fig. 3*E*) and the H2-S3 loop of one α-tubulin, the M loop of the adjacent α-tubulin, and the H1-S2 loop of β-tubulin (Fig. 4 *B–E*). SPACA9 also interacts with two residues, D33 and Q35, that are upstream of the α-tubulin K40 loop (residues 37 to 47) (Fig. 4*C*). These interactions appear to stabilize the αK40 loop, which is disordered in cryo-EM structures of in vitro–assembled acetylated or deacetylated microtubules (28), but has clear density in the SPACA9-bound structure (Fig. 4*F*). The taxol-binding pocket of β-tubulin, a hotspot for microtubule-stabilizing interactions by MIPs, is not occupied by SPACA9 (Fig. 4*A*).

As MIPs may change the geometry of the microtubules to which they are bound, we compared B tubule protofilaments in the bovine DMT (which lack SPACA9) with the equivalent SPACA9-bound protofilaments in the human DMT (Dataset S2). First, we analyzed the longitudinal spacing between tubulin heterodimers within a protofilament. We found that while there is some variation (<1 Å) between neighboring protofilaments, SPACA9 does not change the longitudinal spacing (*SI Appendix*, Fig. S6*A*). Second, we analyzed whether SPACA9 might widen or compress interprotofilament angles. Again, comparison of equivalent interprotofilament angles of bovine and human DMTs revealed no major differences (*SI Appendix*, Fig. S6*B*). These results indicate that SPACA9 binds to but does not remodel the tubulin lattice in the B tubule.

### SPACA9 Forms the TAILS Complex of Human Sperm SMTs.

The 8-nm SPACA9 striations resembled a short version of the TAILS structure observed by cryo-ET in the distal region of mammalian sperm flagella (6, 26). To test the hypothesis that SPACA9 is responsible for TAILS in human sperm, we used single-particle cryo-EM to visualize the SMTs of the distal tip of partly demembranated but otherwise intact human sperm flagella (Fig. 1, *Bottom*). We were able to obtain a 6-Å resolution consensus structure of a sperm SMT that could be improved to 4.5 Å using focused refinements with local masks applied to protofilament pairs (*SI Appendix*, Fig. S1 *C* and *D* and S2 *F–H*).

**Fig. 5.**
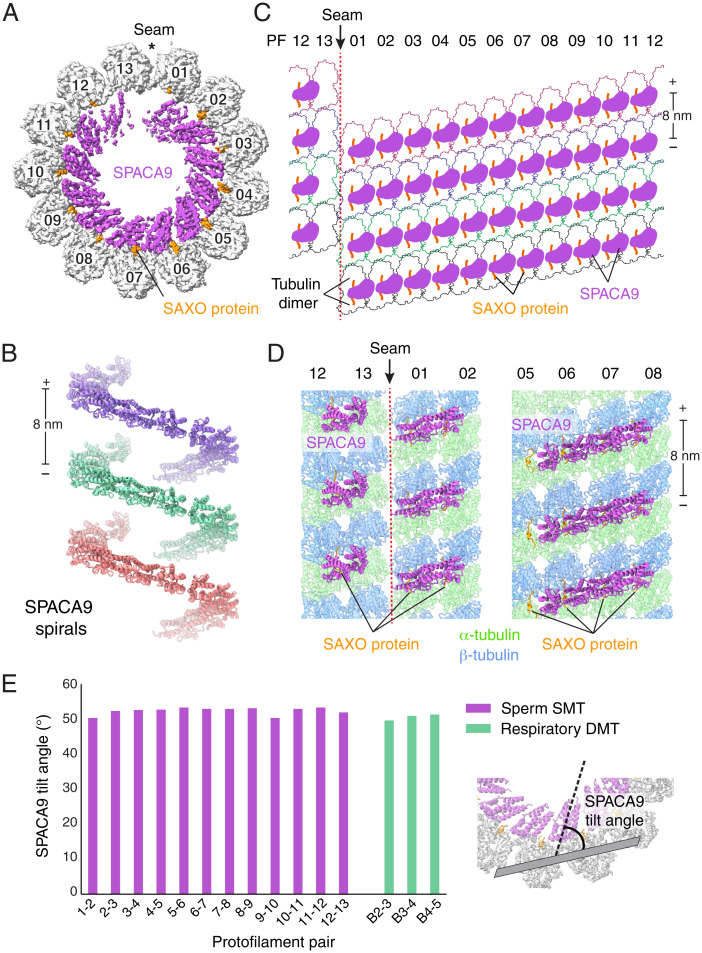
SPACA9 forms spirals in human sperm flagella SMTs. (*A*) Cross-section of the cryo-EM map of a SMT from the distal region of human sperm flagella showing an intralumenal TAILS structure formed by 12 copies of SPACA9 and SAXO proteins. SPACA9 binds to all protofilament pairs, except those at the microtubule seam. (*B*) Longitudinal view of human sperm SMTs with the microtubule removed showing noncontinuous left-handed spirals of SPACA9 separated by 8 nm. The polarity of the microtubule is indicated at the ends of the scale bar. (*C*) Unfurled SMT showing the arrangement of SPACA9 and SAXO proteins in the lumen of the human sperm SMT. (*D*) Atomic models of four protofilament sections showing the arrangement of SPACA9 and SAXO proteins on protofilaments near (*Left*) and opposite (*Right*) the seam. (*E*) Plot of the tilt angles of SPACA9 relative to the protofilament pairs to which they bound. The tilt angle was calculated between the long axis of SPACA9 (shown as dashed line) and the plane of the two tubulin dimers with which it interacts (shown in gray) using the “define” and “angle” commands in Chimera (29).

The structure of the sperm SMT has well-resolved density for the TAILS complex (Fig. 5*A*). At the low resolution provided by cryo-ET, it had not been possible to distinguish whether TAILS was formed by a single large C-shaped monomer or a polymeric assembly. Our structure demonstrates that each spiral unit of TAILS is formed by 12 copies of SPACA9. The localization of SPACA9 in respiratory and sperm ciliary microtubules is consistent with its expression across multiple ciliated tissues in humans (30). Neighboring TAILS are 8 nm apart along the longitudinal axis of the microtubule, consistent with the 8-nm periodicity of tubulin dimers (Fig. 5*B*). As SPACA9 binds the interface between protofilaments, it cannot recognize the heterotypic lateral interactions between α- and β-tubulin subunits that occur at the seam (Fig. 5 *C* and *D*), which explains why TAILS is not a continuous spiral but stops at the seam (6). The SPACA9 densities on either side of the seam are less well resolved than the central copies (Fig. 5*A*), consistent with our observation from the human respiratory DMT that neighboring molecules appear to enhance stability.

The TAILS complex was present on all of the SMTs particles examined. This observation excludes the possibility that preexisting SPACA9 in the B tubule is necessary to initiate TAILS formation, as this would result in only half of the SMTs (those derived from a B tubule) having TAILS. Although TAILS may grow from the arcs of SPACA9 in the B tubule, a separate mechanism must occur for A tubule–derived SMTs. The absence of conventional DMT MIPs in any of the SMTs suggests that they are either displaced by SPACA9 or that their binding is dependent on a preexisting DMT arrangement or that their presence nucleates formation of a DMT, as previously hypothesized (10). Notably, we did not observe any SMTs with TAILS-like densities in our analysis of human respiratory axonemes.

To determine whether the conformation of SPACA9 is conserved within and between respiratory DMTs and sperm SMTs, we calculated the tilt angle for each SPACA9 molecule relative to the microtubule. All of the tilt angles are similar, ranging between 50° and 53° (Fig. 5*E*). Moreover, the 3 SPACA9 proteins of the respiratory DMT striations and 12 SPACA9 proteins of the sperm SMT spirals have a root-mean-square deviation of 0.6 to 1.9 Å after superposing their associated α-tubulins. These measurements demonstrate that SPACA9 bind protofilaments in a similar fashion in different microtubules regardless of the higher order structures they form.

Although our analysis of SPACA9 in the B tubules of respiratory DMTs did not indicate a role for SPACA9 in altering microtubule geometry (*SI Appendix*, Fig. S6 *A* and *B*), we compared the interprotofilament angles of SPACA9-bound sperm SMT with in vitro–assembled undecorated microtubules (31). As expected, protofilament pairs have similar angles in the two microtubules (*SI Appendix*, Fig. S6*C*). The only difference was at the microtubule seam, where the interprotofilament angle in the sperm SMT is more similar to the nonseam positions, suggesting that the spiral organization of SPACA9 may render microtubules more circular.

### SPACA9 Binds in Conjunction with a SAXO Protein.

Our cryo-EM maps revealed that SPACA9 binds respiratory DMTs and sperm SMTs in conjunction with one or more SAXO proteins (Figs. 3*A* and 5*A*). SAXO proteins (short for stabilizer of axonemal microtubules) are MIPs that bind in the longitudinal direction along protofilaments. They are characterized by multiple Mn motifs, which are short α helices that bind at the intradimer interface between α- and β-tubulin and are spaced 8 nm apart (32). SAXO proteins have also been observed on DMTs (10) and CA microtubules (18). In our structures, each SPACA9 molecule interacts with two parallel Mn motifs on adjacent protofilaments (Fig. 4*A*). Specifically, SPACA9 interacts with one Mn motif using two conserved residues, Q20 and Y117, and with the Mn motif of another molecule using the conserved residue, H37 (Fig. 4 *B–E*).

As Mn repeats have similar sequences, and their densities are averages after applying 8-nm and 48-nm periodicity (for the SMT and DMT reconstructions, respectively), we cannot identify the individual SAXO protein(s) from the cryo-EM density, although main chains were traced. The most likely candidates are SAXO1 and SAXO2 (33), which are present only in ciliated eukaryotes. In humans, these proteins share 34% identity, 53% similarity, and each have ≥10 Mn motifs. Despite their similarity, SAXO1 and SAXO2 have distinct patterns of tissue expression in humans. SAXO1 is highly enriched in testes (33), present in human sperm proteomes (34, 35), and has been localized along the length of spermatozoan axonemes by immunofluorescence and immunogold labeling (33). Unlike SAXO1 (33), SAXO2 and its transcripts have been found in the multiciliated cells of differentiated airway epithelia by single-cell RNA sequencing (36) and in human airway cilia by mass spectrometry (25). These lines of evidence suggest that SAXO1 interacts with human sperm axonemes and SAXO2 with respiratory cilia axonemes, but further work will be required to identify whether it is these SAXO proteins that colocalize with SPACA9 in the ciliary microtubules examined here.

### TAILS Resembles the MIP Organization of Apicomplexan Cortical Microtubules.

The arrangement of SPACA9 and SAXO proteins in sperm SMTs is reminiscent of the spiral arrangement of MIPs in the cortical microtubules of the apicomplexan human pathogen *Toxoplasma gondii* (32, 37) (*SI Appendix*, Fig. S7 *A* and *B*). In cortical microtubules, the SAXO protein is SPM1, and the spiral is made up of two closely related paralogs, TrxL1 and TrxL2 (32). Like SPACA9, these proteins recognize interprotofilament interfaces and cannot bind the microtubule seam, leading to interrupted spirals that follow the pseudohelical symmetry of the microtubule. Despite these similarities, TrxL1 and TrxL2 have a thioredoxin-like fold that is unlike the helical fold of SPACA9 (*SI Appendix*, Fig. S7*C*). It is therefore probable that these proteins evolved separately to engage microtubules in similar ways, and as a result may perform similar functions. The MIPs of *Toxoplasma gondii* have been shown to stabilize cortical microtubules in response to chemical stress (32). In *Toxoplasma gondii,* the localization of TrxL1 or TrxL2 to cortical microtubules is dependent on SPM1 (32, 38), suggesting that the SAXO proteins observed in human SMTs and DMTs may be required for the recruitment of SPACA9 to ciliary microtubules.

Unlike the generally circular SPACA9-bound sperm SMT, the interprotofilament angles of *Toxoplasma gondii* cortical microtubules vary dramatically among the 10 TrxL1-bound regions (23° to 28°), the 2 TrxL2-bound regions (37° to 39°), and the seam (22°) (*SI Appendix*, Figs. S6*C* and S7). This difference in microtubule roundness indicates that although TrxL1/L2 and SPACA9 form similar intralumenal spirals, they have different effects on microtubule geometry.

### Conservation of Lumenal Striations of Animal Sperm SMTs.

**Fig. 6.**
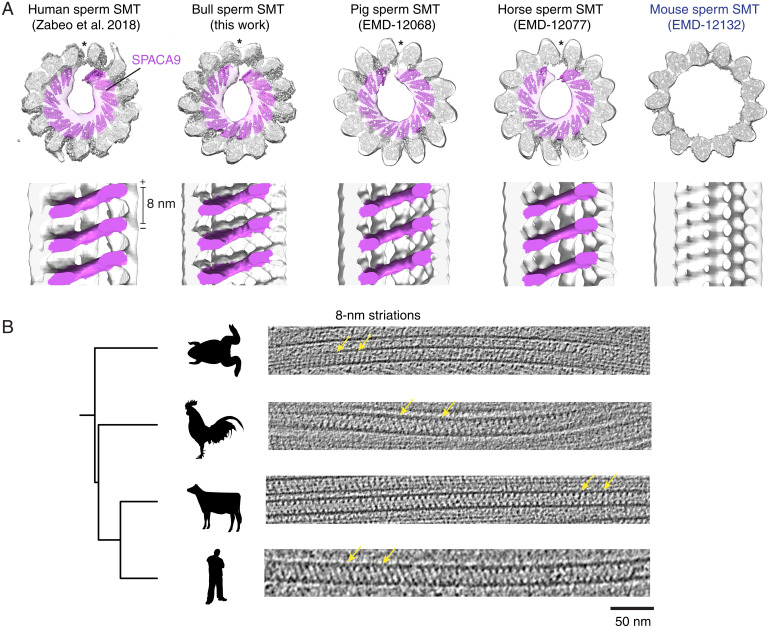
Conservation of SPACA9-like striations in animal sperm ciliary SMTs. (*A*) Cross-section (*Top*) and longitudinal slices (*Bottom*) of subtomogram averages of human, bull, pig, horse, and mouse (from *Left* to *Right*) sperm SMTs with the atomic model of the human sperm SMT fitted inside. Microtubule seams are indicated with an asterisk, and microtubule polarity is indicated on the scale bar. The lumen of the mouse sperm SMT lacks SPACA9-like densities. (*B*) A phylogenetic tree showing slices of tomograms of SMTs from the flagella tips of frog, chicken, bull, and human (from *Top* to *Bottom*). Yellow arrows point to 8-nm repeating striations. XYZ views of the tomograms are provided in *SI Appendix*, Fig. S8.

To determine whether TAILS is conserved in the SMTs of other species sperm, we compared our atomic model of human sperm SMT with cryo-ET subtomogram averages of pig, horse, and mouse sperm SMTs (26). While our model agrees well with the subtomogram averages of pig and horse sperm SMTs, mouse sperm lack TAILS (Fig. 6*A*). To achieve a better understanding of whether mice are outliers among animals, we used cryo-ET to visualize intact spermatozoa from bull (*Bos taurus*), chicken (*Gallus gallus domesticus*), and the western clawed frog (*Xenopus tropicalis*). The tomograms revealed that each contain striations reminiscent of SPACA9 (Fig. 6*B* and *SI Appendix*, Fig. S8). However, for chicken sperm, these striations existed only in distal DMTs, as their flagella lacked a singlet zone and had instead a disordered array of DMTs that terminated sporadically but completely. In frog sperm, 8-nm striations were found in both SMTs and the B tubule of DMTs (Fig. 6*B*). In contrast to other species, the striations were not present in all of the SMTs analyzed; frog microtubules without internal striations were sometimes observed as well. Frog sperm was the only sample obtained from the dissection of testes rather than ejaculate, so it is unclear whether this observation reflects a difference between species or sperm maturity. Collectively, these results demonstrate that although not universal, microtubules with lumenal striations are commonly found in animal respiratory and sperm SMTs and DMTs. Consistent with TAILS only being present in organisms encoding SPACA9, we did not observe striated microtubules in the flagella of the protist *T. brucei* after analyzing previously determined tomograms (5, 39) or in tomograms of the ciliary tip of *C. reinhardtii* (40).

## Discussion

Here, we have resolved the structure of the DMT from human respiratory cilia and built atomic models for the 33 proteins that associate with its lumenal surfaces. Among these MIPs, 6 were previously unknown to bind mammalian DMTs (CFAP68, CFAP77, CFAP90, FAM183, FAM166C, and SPACA9). Using single-particle analysis cryo-EM, we revealed that one of these MIPs, SPACA9, is also the main component of the TAILS complex found in the distal SMTs of human spermatozoa.

Despite the presence of SPACA9 on two different human ciliary microtubules, its function remains unclear. A mouse knockout model is viable, fertile, and has no clear phenotypic differences from wild-type mice (41). However, mice appear to lack both TAILS in sperm SMTs (Fig. 6*A*) and SPACA9-like decorations in the B tubule of sperm DMTs (*SI Appendix*, Fig. S5*B*), making it a poor model organism for studying SPACA9 function. The absence of SPACA9 in mouse sperm SMTs does, however, argue against the protein functioning in the splitting of DMTs into SMTs. We propose that SPACA9 and the SAXO protein(s) with which it colocalizes stabilize the protofilaments to which they are bound, particularly against external stress. This proposed function agrees with the ability of SPACA9 and its SAXO protein to bind across tubulin interfaces, the general property of Mn motif-containing proteins to stabilize microtubules against depolymerization (33, 42), and the stabilizing function of TAILS-like cortical MIPs in *Toxoplasma gondii* (32). A general ability to stabilize the microtubule lattice may explain the distribution of SPACA9 on different ciliary microtubules. Differences in SPACA9 distributions between cell types (e.g., in the distal SMTs of spermatozoa that are absent in human airway cilia) may generate cell-specific susceptibilities to SPACA9 loss.

The crisscross relationship between SPACA9 and the SAXO proteins on the microtubule lattice (with SPACA9 binding in the lateral direction and SAXO proteins in the longitudinal direction) is replicated by MIPs in all of the ciliary microtubules for which we have detailed atomic models. DMTs combine SAXO proteins and filamentous MIPs with protofilament-spanning lateral proteins such as EFHC1 (10, 11), whereas CA microtubules have SAXO proteins and arc-MIPs (18). Intersecting MIPs are also found in the cortical microtubules of parasites such as *Toxoplasma gondii* (32) in the form of SAXO proteins and TrxL1/2. The orthotropic properties provided by this conserved combination of MIPs could provide stability to the microtubule lattice from forces operating in multiple directions.

Mammalian primary cilia have extensive singlet zones, but no TAILS-like structures (2, 3). Instead, the external microtubule lattice is bound by an end-binding (EB)–like protein (3). Apart from binding the external rather than the lumenal surface, the EB-like protein and SPACA9 share many characteristics: both have 8-nm periodicity, both follow the pseudohelical path of the microtubule, both bind interfaces between tubulin monomers, and neither are present at the seam. It is therefore possible that the EB-like protein of primary cilia and SPACA9 of motile cilia have similar stabilizing functions.

Interestingly, the distinctive patterns made by SPACA9 (8-nm repeating striations in the DMT B tubule and incomplete spirals in SMTs) are observed in other microtubules of the cilium-centrosome complex. Cryo-ET of the transition zone, the region between the basal body and the axoneme, of bovine trachea cilia has revealed two striations in the A tubule and one in the B tubule (20) (*SI Appendix*, Fig. S9*A*). Although these densities have a size and shape similar to those of SPACA9 striations, the low resolution of the subtomogram average prevents a conclusive assignment, and other proteins could generate similar appearing densities. A number of 8-nm repeating lumenal striations are also found within the TMTs of the pig sperm proximal centriole (26) (*SI Appendix*, Fig. S9*B*) and on the CA microtubules of human (6), horse, and pig sperm axonemes (26) (*SI Appendix*, Fig. S9*C*). If these densities do correspond to SPACA9, then it would indicate that SPACA9 is a polyspecific MIP of almost every ciliary microtubule in both sperm and respiratory cilia. This would contrast with our prior understanding of MIPs from *C. reinhardtii*, which are exclusive for particular microtubules (10, 18, 19). High-resolution structural information of other ciliary microtubules will be necessary to confirm whether SPACA9 is indeed a promiscuous MIP.

In summary, the identification of the proteins that bind human ciliary microtubules and their spatial distribution within the cilium is a step toward understanding the complex biogenesis of the axoneme. As mutations in some microtubule-bound axonemal components can affect sperm motility but leave respiratory cilia function unchanged (16), our structures will provide better references to understand and interpret disease-causing mutations that affect ciliary MIPs or tubulin isotypes. Furthermore, our demonstration that single-particle cryo-EM methods can be applied to human ciliary microtubules provides a tool for the analysis of samples obtained directly from ciliopathy patients.

## Materials and Methods

### Human Subjects.

Human respiratory epithelial cells were collected from the inferior nasal turbinate of a healthy volunteer at the Royal Brompton Hospital (London, UK) by nasal scrape biopsy. The subject provided informed consent for use of their cells for research. This study was approved by the Health Research Authority Bloomsbury Research Ethics Committee (ref. 08/H0713/82). Human sperm cells were obtained from semen donated by healthy men at the Male Fertility Lab at the University of Washington Medical Center. Semen samples were collected from consenting donors and provided anonymously to this study as approved by University of Washington institutional review board protocol no. 51899.

### Culturing of Human Airway Epithelial Cells.

Nasal biopsies were performed with a 3-mm cytology brush (ConMed). Cells were dissociated from the brush by gentle agitation in PneumaCult-Ex Plus medium (STEMCELL Technologies, cat. no. 05040). The cells were seeded into a single well of a culture plate coated in collagen (PureCol, Sigma-Aldrich, cat. no. 5006–15MG). Once confluent, the cells were passaged and expanded further in a T25 flask. The cells were passaged a second time and seeded onto transwell inserts (Corning, cat. no. CLS3460) at a density of 24,000 cells per insert. Cells were cultured in PneumaCult-Ex Plus medium until confluent, at which time the media in the basal chamber was replaced with PneumaCult-ALI medium (STEMCELL Technologies, cat. no. 05001) and the apical surface exposed to provide an ALI. All media contained Primocin (InvivoGen, cat. no. ant-pm-05) and penicillin-streptomycin (Thermo Fisher Scientific, cat. no. 15070063) to prevent bacteria growth. Ciliation was observed 4 to 6 wk after transition to ALI. During differentiation, the basolateral media was refreshed every 2 to 3 d and the apical surfaces were washed with phosphate-buffered saline (PBS) to remove mucus.

### Isolation of Human Respiratory Axonemes.

Ciliary axonemes were isolated from well-differentiated ALI cultures as described (21). In brief, ciliated cell surfaces were washed twice with PBS for 5 min to remove cell debris and mucus. Ice-cold PBS was then added to both compartments of the culture dish and the dish placed on ice. After 5 min, the PBS solution was removed and 50 μL ice-cold deciliation buffer (10 mM Tris, pH 7.5, 50 mM NaCl, 10 mM CaCl_2_, 1 mM EDTA, 0.1% Triton X-100, 7 mM β-mercaptoethanol, 1% protease inhibitor mixture [Sigma-Aldrich, cat. no. P8340]) was added to the cells of each well. After a 2-min incubation without shaking, the cilia-containing solution was transferred to a microcentrifuge tube. Cellular debris and mucus were removed by centrifugation at 1,000 × *g* for 1 min at 4 °C. Axonemes were collected by centrifuging the supernatant at 15,000 × *g* for 5 min at 4 °C. The axonemal pellet was resuspended in buffer (30 mM HEPES pH 7.3, 1 mM ethylene glycol tetraacetic acid, 5 mM MgSO_4_, 0.1 mM ethylenediaminetetraacetate [EDTA], 25 mM NaCl, 1 mM dithiothreitol, 1% protease inhibitor mixture, and 100 g/mL soybean trypsin inhibitor [Sigma-Aldrich, cat. no. T9128]), flash-frozen in liquid nitrogen, and stored at −80 °C before use.

### Purification of Human Spermatozoa.

Motile human spermatozoa were purified using the swim-up method. Semen from healthy individuals was incubated at 37 °C for at least 20 min to allow liquefaction, then was gently mixed and placed under 3 mL HTF-HSA media (human tubal fluid supplemented with 0.5% human serum albumin) in a 15-mL conical tube. The tube was then placed in a rack at a 30° angle, avoiding mixture of the 2 layers, and incubated under 5% CO_2_ for 1 h at 37 °C. The supernatant layer was aspirated without disturbing the semen layer, and spermatozoa were examined for motility by light microscopy. This layer was then subjected to centrifugation at 250 × *g* for 5 min. The supernatant was discarded, and the pellet resuspended in Hank’s balanced salt solution (HBSS) and brought to a concentration of 10 million cells per milliliter.

### Preparation of Animal Spermatozoa.

Animal sperm samples were provided as gifts. *B. taurus* (bull) spermatozoa from ejaculate were provided by Jonas Krantz (VikingGenetics). *G. gallus* (chicken) spermatozoa from ejaculate were provided by Robin Abbey-Lee, Fabio Pértille, and Enya van Poucke (Linköping University). *X. tropicalis* (frog) spermatozoa from dissected testes were provided by Simone Reber (Humboldt University of Berlin).

### Mass Spectrometry Analysis.

Isolated human respiratory axonemes were sent for mass spectrometry analysis at the Taplin Mass Spectrometry Facility at Harvard Medical School as an excised and dehydrated sodium dodecyl sulfate-polyacrylamide gel electrophoresis gel piece. The gel piece was rehydrated with 50 mM ammonium bicarbonate solution containing 12.5 ng/mL trypsin (Promega, cat. no. 90057). After 45 min at 4 °C, the trypsin solution was replaced with 50 mM ammonium bicarbonate solution and left at 37 °C overnight. Peptides were extracted by removing the ammonium bicarbonate solution, followed by a wash with a solution containing 50% acetonitrile and 1% formic acid. The extracts were then dried using a vacuum concentrator for 1 h and stored at 4 °C. For mass spectrometry, the samples were reconstituted in 5 to 10 mL of solvent A (2.5% acetonitrile, 0.1% formic acid) and loaded onto a pre-equilibrated reverse-phase capillary column (100 mm inner diameter and ∼30 cm length) containing 2.6 mm C18 spherical silica beads using a Famos auto sampler (LC Packings). A gradient was formed, and peptides were eluted with increasing concentrations of solvent B (97.5% acetonitrile, 0.1% formic acid). As peptides eluted, they were subjected to electrospray ionization and entered into an LTQ Orbitrap Velos Pro ion-trap mass spectrometer (Thermo Fisher Scientific). Peptides were detected, isolated, and fragmented to produce a tandem mass spectrum of fragment ions for each peptide. Protein identity was determined from the acquired fragmentation pattern using Sequest (Thermo Fisher Scientific). The data were filtered to between a 1 and 2% peptide false discovery rate. The results of the analysis are provided in Dataset S1.

### Negative-Stain EM.

Negative-stain EM was used to monitor the purification of axonemes from ALI cultures. A 4-μL aliquot of sample was applied to a glow-discharged continuous carbon grid (EMS, cat. no. CF200-Cu). The sample was incubated on the grid for 1 min and excess sample was removed with filter paper. The grid was washed twice and incubated for 2 min with 4 μL of 1.5% uranyl formate solution, followed by blotting to remove excess solution. After air-drying at room temperature, the grid was examined with a Tecnai T12 electron microscope (Thermo Fisher Scientific) operating at 120 kV and equipped with a UltraScan 895 camera (Gatan) at Harvard Medical School. An example micrograph of an isolated axoneme is provided in Fig. 1.

Negative-stain EM was also used to optimize the demembranation of mammalian sperm. Bovine ejaculate was washed by centrifugation at 500 × *g* and resuspended in an equal volume of HBSS 3 times, with the final resuspension in demembranation buffer (HBSS plus 50 mM KCl and 0.25% Triton X-100). Cells were incubated in demembranation buffer for 20 min, and 5 μL of this mixture was applied to a glow-discharged 300-mesh copper grid with carbon film (EMS, cat. no. CF300-Cu). After a 1-min incubation, the grid was blotted and applied to a drop of 2% uranyl acetate for 30 s and then blotted, then once again applied to a drop of 2% uranyl acetate for 30 s and blotted. Air-dried grids were imaged with a Tecnai Spirit T12 electron microscope (FEI) at the University of Gothenburg. An example micrograph of a demembranated and splayed sperm axoneme is provided in Fig. 1.

### Preparation of Samples for Cryo-EM.

To make cryo-grids for single-particle analysis of human respiratory DMTs, 3 μL axonemes with an absorbance reading at 280 nm of 5.8 was applied to glow-discharged Quantifoil holey carbon grids (R2/1, copper, 400 mesh, Quantifoil Micro Tools, cat. no. Q410CR1). The grids were blotted for 10 to 11 s with a blot force of 10 in 100% humidity before being plunged into liquid ethane cooled by liquid nitrogen by using a Vitrobot Mark IV (Thermo Fisher Scientific).

For single-particle analysis of human sperm microtubules, spermatozoa (at ∼10 million cells per milliliter) were mixed with an equal volume of demembranation buffer (HBSS supplemented with 50 mM KCl, 0.25% Triton X-100, and 1 mM phenylmethanesulfonylfluoride) and incubated for 20 min before grid making. A total of 4 µL of sample was applied to 200-mesh lacey carbon grids (EMS, cat. no. LC200-Cu) at 100% humidity before plunge freezing into liquid ethane with a Vitrobot Mark IV (Thermo Fisher Scientific). Blotting times ranged from 8 to 10 s, with a blot force of 5.

To make cryo-grids for cryo-ET analysis, animal sperm samples were plunge frozen with a Leica GP (Leica Microsystems). *B. taurus* spermatozoa were prepared within 24 h of collection by centrifuging ejaculate at 300 × *g* for 8 min, resuspending the cells in PBS, centrifuging once again, and resuspending in an equal volume of PBS. *G. gallus* spermatozoa were prepared by diluting ejaculate in Dulbecco’s modified Eagle’s medium at ratios ranging from 1:100 and 1:1,000 immediately after collection and plunge freezing ∼6 h after collection. *X. tropicalis* spermatozoa were diluted 1:100 in HBSS and plunge frozen immediately after dissection from testes. For all of the species listed above, 5 µL of sample were applied to lacey carbon grids (Ted Pella, cat. no. 01894-F) and blotted for 4 to 6 s at 30% humidity.

### Cryo-EM Screening.

Cryo-grids of human respiratory axonemes were screened with either a Tecnai F20 microscope (Thermo Fisher Scientific), equipped with a K2 Summit detector (Gatan), or a Talos Arctica microscope (Thermo Fisher Scientific) equipped with a K3 detector (Gatan) at the Harvard Cryo-EM Center for Structural Biology. Cryo-grids of human sperm samples were screened on a Glacios microscope (Thermo Fisher Scientific) equipped with a K2 Summit detector (Gatan) at the Arnold and Mabel Beckman Cryo-EM Center at the University of Washington. Cryo-grids of animal sperm were screened on an FEI Tecnai Spirit T12 microscope (FEI) at the University of Gothenburg.

### Cryo-EM Data Collection.

#### Human Respiratory DMTs.

Data acquisition of human respiratory cilia microtubules for single-particle analysis was performed at the Harvard Cryo-EM Center for Structural Biology. Images were acquired on a Titan Krios microscope (Thermo Fisher Scientific) equipped with a BioQuantum K3 imaging filter (slit width, 25 eV) and a K3 detector (Gatan). Images were recorded at a defocus range of −0.8 to −2 μm with a nominal magnification of 64,000× in counting mode, resulting in a pixel size of 1.37 Å. Each image was dose fractionated into 50 movie frames with a total exposure time of 3.76 s and a total electron dose of ∼60 electrons per square Angstrom. Images were collected in 9 holes per stage move with 3 shots per hole. A total of 37,071 movies were collected using SerialEM version 3.8.6 (43) from 2 independent data collection sessions. An example micrograph is provided in Fig. 1.

#### Human Sperm Microtubules.

Data acquisition of human sperm microtubules for single-particle analysis was performed at the Arnold and Mabel Beckman Center for Cryo-EM at the University of Washington. Images were acquired on a Titan Krios equipped with a Gatan K3 detector and imaging filter (slit width, 25 eV). Images were recorded at a defocus range of −1 to −3 μm with a nominal magnification of 105,000× in superresolution mode, resulting in a pixel size of 0.4215 Å. Data were collected at a rate of 25 frames per second with a total exposure time of 3 s and dose of 40 electrons per square Angstrom. A total of 1,232 movies were collected using Leginon (44). An example micrograph is provided in Fig. 1.

#### Animal Sperm.

Tilt series of spermatozoa from *X. tropicalis*, *G. gallus*, and *B. taurus* were collected on a Titan Krios microscope equipped with a Gatan K2 BioQuantum camera and energy filter at the Umeå Core Facility Electron Microscopy (UCEM). A defocus range of −4 to −6 μm was used and the acquisition was performed with Tomography version 5.2.0 (Thermo Fisher Scientific). Data were collected in counting mode with a nominal magnification of 33,000× following a dose symmetric tilt scheme (45) and a total electron dose of 100 electrons per square Angstrom (or 81 electrons per square Angstrom for chicken sperm) spread over 61 tilt images (0° ± 60° every 2°), with a pixel size of 4.37 Å. Each tilt image was generated by applying motion correction to 8 frames (0.2 electrons per square Angstrom each) with MotionCor2 (46).

### Image Processing.

#### Single-Particle Analysis of Human Respiratory DMTs.

A diagram summarizing the processing of human respiratory DMTs is shown in *SI Appendix*, Fig. S1*B*. All image processing was performed using RELION-3.1 (47) or RELION-4.0 (48) unless otherwise stated. During image import, distinct optics groups were assigned to images collected with different image shifts, resulting in 54 (27 × 2 sessions) optics groups. A total of 37,071 movie stacks collected using counting mode were motion corrected and electron-dose weighted using MotionCor2 (46). CTFFIND4 (49) was used to calculate contrast transfer function (CTF) parameters from the motion-corrected micrographs. A total of 16,933 micrographs were selected for further processing after excluding micrographs that lacked microtubules or had poor Thon ring fitting from CTFFIND4. To pick particles, start points and endpoints for the microtubules were selected using RELION manual picking. Particles were extracted with a helical rise of 8.2 nm and the number of unique asymmetric units set to 1, resulting in 2,582,833 particles of the 8-nm repeat. Particles were extracted in 512-pixel boxes, downscaled to 256-pixel boxes to accelerate computation. Two-dimensional (2D) classification was performed only to check image quality, not to exclude particles (*SI Appendix*, Fig. S1*A*). The map of the bovine respiratory DMT (EMD-24664) (11), low-pass filtered to 15 Å, was used as an initial reference for 3D refinement, followed by 3D classification without image alignment to exclude bad particles. To increase the number of retained particles, we repeated the process and merged all good particles while excluding duplicates. In total, 1,903,665 “8-nm particles” were retained and re-extracted without binning. The 16-nm particles were generated from the 8-nm particles using 3D classification with a cylinder mask on 16-nm repeating MIPs near the inner junction. The 48-nm particles were generated from the 16-nm particles using 3D classification with a cylinder mask on 48-nm repeating MIPs near the seam of the A tubule. Following classification, a total of 208,558 particles were subjected to additional rounds of 3D refinement, CTF refinement, and Bayesian polishing. The final resolution of the “consensus map” was 3.6 Å. To improve map quality further, we used local refinement strategies (10, 11). Briefly, we generated 30 overlapping cylindric masks, each covering 2 or 3 protofilaments and one-third of the longitudinal axis of the DMT, and performed local refinements one mask at a time. The resolution within each mask improved to 3.1 to 3.5 Å. Mask-focused classification was performed on the SPACA9-bound region of the B tubule and the tektin bundle of the A tubule. Specifically, we observed classes corresponding to no tektin (47%), 3 to 4 tektin filaments (33%), and 6 tektin filaments (20%). The map of the 6-filament class was used in the final composite map for model building.

#### Single-Particle Analysis of Human Sperm Microtubles.

A diagram summarizing the processing of human sperm microtubules is provided in *SI Appendix*, Fig. S1*D*. A total of 1,136 superresolution movie stacks, downscaled to 0.843 Å per pixel, were motion corrected using RELION and CTF corrected using CTFFIND4 (49). Microtubules were manually picked from the micrographs in RELION 3.1. A total of 78,359 particles, downscaled to 1.686 Å per pixel to reduce computational costs, were extracted in 400-pixel boxes. Two-dimensional classification was used to clean the dataset, and the retained particles subjected to supervised 3D classification using low-pass filtered reference maps of a DMT and SMT created from the human respiratory DMT. In total, we isolated 36,697 SMT particles. Tubulin density was subtracted, and 3D classification was performed focused on MIPs. Classes were obtained showing TAILS but with misaligned rotations and 4-nm shifts (corresponding to the distance of a single tubulin monomer). The particles were realigned to a common reference using a bespoke script, re-extracted, and subjected to a round of 3D refinement. Unbinned particles (0.843 Å per pixel) were then re-extracted and subjected to 3D classification focused on the MIPs, followed by particle orientation realignment. We CTF refined, polished, and reclassified 21,990 selected particles to provide a 6.0-Å resolution consensus map. Protofilament-focused refinement and classification improved the resolution to 4.5 to 4.7 Å.

For both human respiratory DMTs and sperm SMTs, composite maps for model building, refinement, and deposition were generated from the locally refined maps using the “fit in map” and “vop maximum” commands in Chimera (29). Maps were sharpened using RELION postprocessing, Phenix.auto_sharpen (27), or DeepEMhancer (50). The DeepEMhancer-sharpened maps were used to make figures unless otherwise stated.

#### Tomogram Calculation and Subtomogram Averaging.

Tomograms of animal sperm were calculated using patch tracking and CTF corrected using the IMOD package (51). Subtomogram averaging of bovine ciliary microtubules was performed with PEET (52). Each SMT was modeled in the 10 tomograms selected for averaging, and coordinates of subvolumes were evenly spaced every 8 nm along modeled filaments. Preliminary averages were first generated by aligning subvolumes belonging to each microtubule individually. Then, each microtubule average was manually realigned by rotating it around its y axis to ensure uniform positioning of the microtubule seam (where there is a gap in TAILS), and this rotation was applied to subvolumes. A local refinement was then performed with all subvolumes (14,753 total) to produce the final average.

### Model Building and Refinement.

Model building was performed in Coot version 0.9.4.1 with torsion, planar peptide, *trans* peptide, and Ramachandran restraints applied (53). Model building of the 48-nm repeat of human respiratory DMT was initiated using the atomic model of the 48-nm repeat of bovine respiratory DMT (PDB: 7RRO) (11). The isotypes of α- and β-tubulin (TUBA1A and TUBB4B, respectively) were identified based on side chain density and their abundance in ciliated cells by mass spectrometry (Dataset S1) and single-cell sequencing (36). AlphaFold2 models of human MIPs, obtained from the AlphaFold Protein Structure Database (AlphaFold DB) (22), were superposed onto the models of their bovine orthologs and manually corrected. All 29 MIPs in the bovine respiratory DMT have orthologs in the human respiratory DMT, except for TEKTIP1 and EFCAB6. SPACA9 on protofilaments B02 to B05 was identified based on side chain density and built de novo. Identification of CFAP68 (C11orf1), CFAP77, CFAP90 (C5orf49), FAM166C, and FAM183A was performed by two complementary strategies guided by side chain density (*SI Appendix*, Fig. S3). Briefly, a polyalanine model was manually traced in the density and was used to either automatically search against the PDB or the AlphaFold2 DB by Foldseek (23) or was manually compared with a bespoke Alphafold2 structure library. The bespoke library contained 745 AlphaFold2 models of the proteins identified by mass spectrometry analysis of the human respiratory axoneme (Dataset S1), which was subsequently screened to contain only small proteins with little secondary structure.

Model building of the 8-nm repeat of human sperm SMT was initiated by fitting tubulin from the A tubule of the DMT. The TAILS complex was modeled by fitting individual copies of SPACA9. Density corresponding to SAXO proteins was modeled for visualization and interpretation purposes only; SAXO proteins are not included in the deposited model as their identity is unknown.

The atomic models were refined against their corresponding maps using Phenix.real_space_refine version 1.19.2-4158 (54), with secondary structure, Ramachandran and rotamer restraints applied. Rotamer restraints target was set to fix outliers, hydrogens were turned on, and the weighting of nonbonded restraints was set at 500. Iterative correction with Coot was performed between rounds of Phenix refinement.

### Structure Analysis and Figures.

SPACA9 tilt angles (Fig. 5*E*) were measured in Chimera. The horizontal plane was generated using the Chimera “define” command and the angle between the SPACA9 long axis and the plane was calculated using the Chimera “angle” command. To calculate the intertubulin dimer distances of DMTs (*SI Appendix*, Fig. S6*A*), the mass center of each tubulin dimer was first calculated using the Chimera “define” command. Each 48-nm protofilament contains 6 tubulin dimers, yielding 5 measurements of intertubulin dimer distances. Interprotofilament angles (*SI Appendix*, Fig. S6 *B* and *C*) were calculated from the atomic models of the human respiratory DMT (this study), bovine respiratory DMT (11), human sperm SMT (this study), in vitro assembled guanosine 5′-diphosphate microtubules (31) and *Toxoplasma gondii* cortical microtubules (32). The interprotofilament angle was calculated based on the relative rotation of tubulin dimers of adjacent protofilaments using the “angle_between_domains” command in PyMOL version 2.3.4 (Schrödinger). The interface area between SPACA9 and tubulin (Fig. 4*A*) was calculated using PDBe PISA version 1.52 (55) and visualized in ChimeraX (56) using the “measure buriedArea” command with a “cutoffArea” of 100. Measurements of sequence conservation (Fig. 3*E*) were performed using the ConSurf Server (57) and visualized in ChimeraX. The sequence alignment in *SI Appendix*, Fig. S4*B* was generated using Clustal Omega version 1.2.2 (58) and visualized in ESPrint version 3.0 (59). The phylogenetic tree (*SI Appendix*, Fig. S4*A*) was extracted from EggNOG version 5.0.0 (accession number ENOG502QQV9) (60) and visualized by iTOL version 6.5.2 (61). Figure panels depicting cryo-EM maps or atomic models were generated with Chimera (29) or ChimeraX (56). The software was installed and configured by SBGrid (62).

### Quantification and Statistical Analysis.

Resolution estimations of cryo-EM density maps are based on the 0.143 Fourier shell correlation criterion (63). All statistical analysis performed on the deposited model (PDB: 7UNG and 7UN1) was done using MolProbity (64) integrated in the PHENIX package (*SI Appendix*, Table S1).

## Supplementary Material

Supplementary File

Supplementary File

Supplementary File

## Data Availability

The composite cryo-EM map of the 48-nm repeat of the human respiratory cilia DMT has been deposited in the Electron Microscopy Data Bank (EMDB) with accession code EMD-26624 (65). The composite cryo-EM map of the 8-nm repeat of the human sperm flagella SMT has been deposited with accession code EMD-26611 (66). Consensus maps, masks, and locally refined maps have been deposited as additional files associated with these EMDB entries. Representative tomograms of frog, chicken, bovine, and human sperm distal microtubules have been deposited with accession codes EMD-14732 (67), EMD-14730 (68), EMD-14723 (69), and EMD-14734 (70), respectively. Subtomogram averages of the human and bull sperm SMT have been deposited to the EMDB with accession codes EMD-15450 (71) and EMD-15451 (72), respectively. The atomic models of the 48-nm repeat of the DMT from human respiratory cilia and 8-nm repeat of the SMT from human sperm flagella have been deposited in the PDB with the accession numbers 7UNG (73) and 7UN1 (74), respectively. The python script used to rotate microtubule particles to align the seam is available at https://github.com/MiaoGui/Microtubule_processing (75).
