# Supplementary material for: SPACA9 is a lumenal protein of human ciliary singlet and doublet microtubules
Source: Proc Natl Acad Sci U S A. 2022 Oct 3;119(41):e2207605119. doi: 10.1073/pnas.2207605119 (PMC9564825; doi:10.1073/pnas.2207605119)
Supplement: Supplementary File [file pnas.2207605119.sapp.pdf]

PNAS

Supporting Information for
SPACA9 is a lumenal protein of human ciliary singlet and doublet microtubules.

Miao Gui, Jacob T. Croft, Davide Zabeo, Vajradhar Acharya, Justin M. Kollman, Thomas Burgoyne, Johanna L. Höög[#], Alan Brown[#]

[#]Correspondence: johanna.hoog@gu.se (J.L.H.); alan_brown@hms.harvard.edu (A.B.)

This PDF file includes:

Figures S1 to S9
Tables S1 to S2
Legends for Datasets S1 to S2
SI References

SI Figures

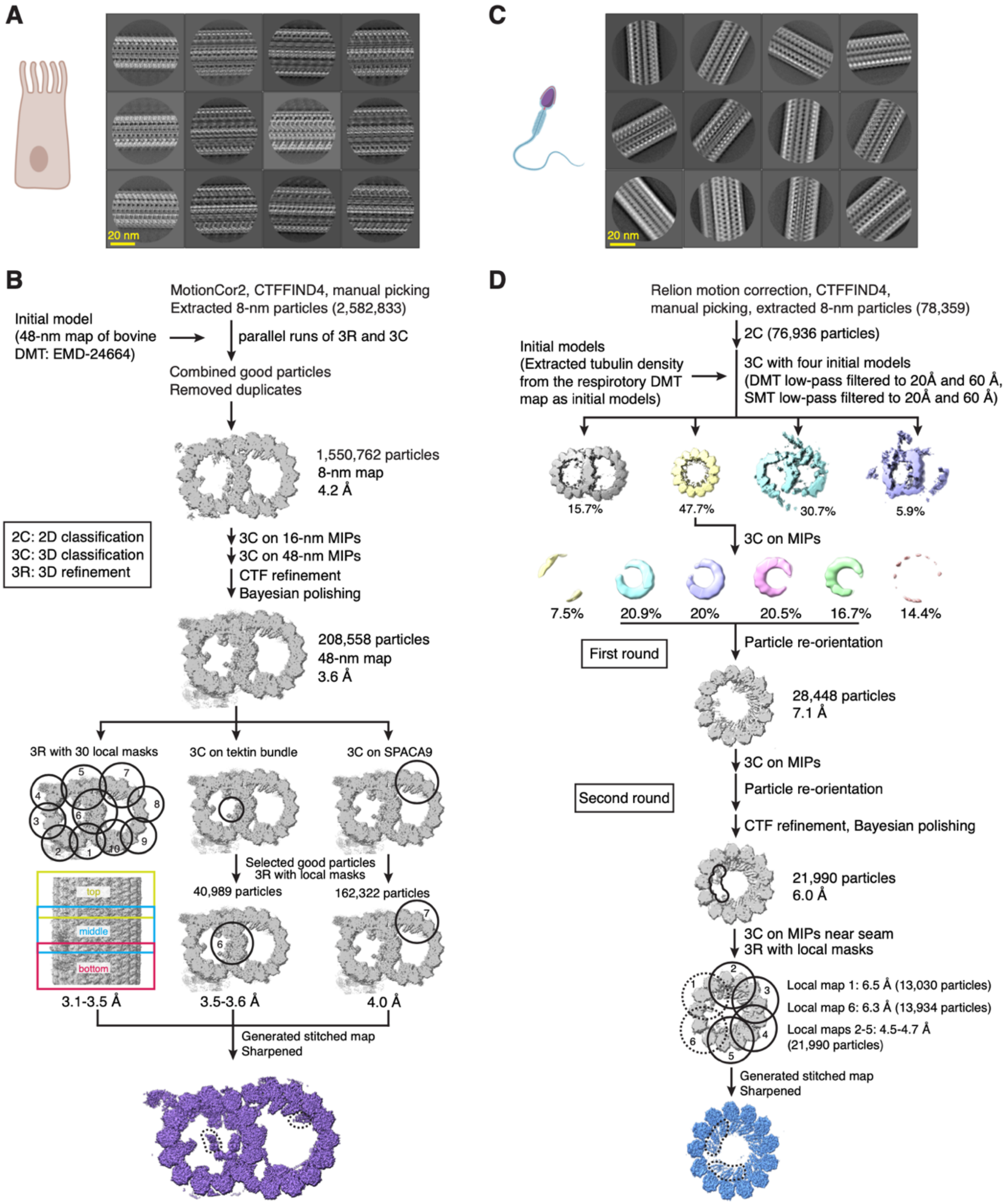

Fig. S1. Processing schemes for human ciliary microtubules. (*A*) Two-dimensional (2D) class averages of doublet microtubules (DMTs) isolated from cultured human respiratory epithelial. (*B*) Processing of human respiratory DMTs. (*C*) 2D classes averages of DMTs and singlet microtubules (SMTs) visible in electron micrographs of frozen hydrated demembranated human spermatozoa. (*D*) Processing of human sperm ciliary microtubules. In panels (B) and (D), dashed outlines within the final maps indicate regions of lower resolution. These regions were modeled without side chains in the final deposited atomic model.

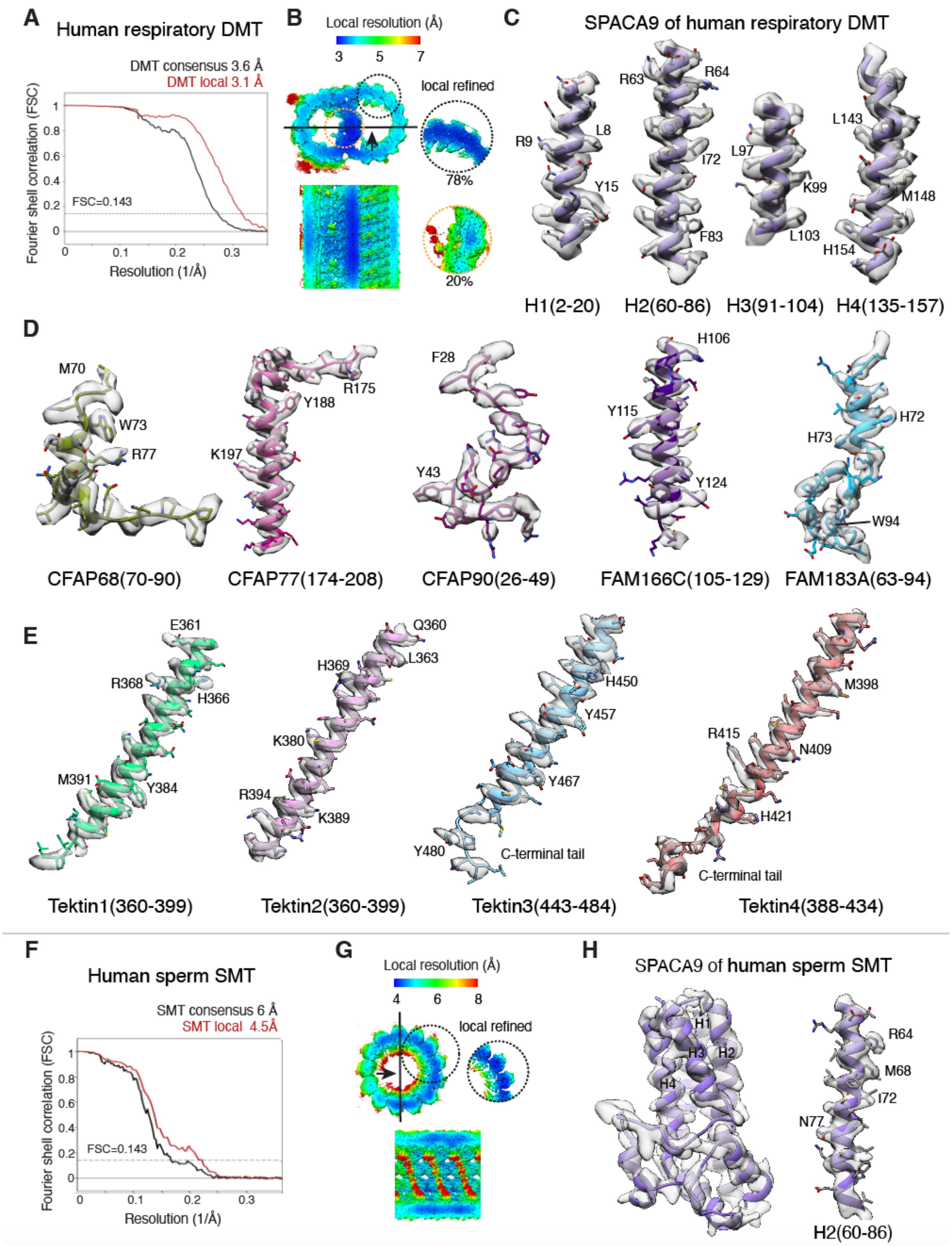

Fig. S2. Global and local resolution. (*A*) Fourier Shell Correlation (FSC) curves for the consensus reconstruction of the 48-nm repeat of the human respiratory doublet microtubule (DMT consensus, black), and the best resolved reconstruction after applying a two-protofilament mask (DMT local, red). (*B*) Map of the DMT consensus refinement colored by local resolution in

cross-section (top) and longitudinal (bottom) view. The circular insets show the improvement in local resolution following focused 3D classification and refinement. Particle percentages from the focused classifications are provided below. (*C*) Model and density for Helices H1–4 (from left to right) of SPACA9 in the human respiratory DMT. (*D*) Model and density for regions of the five other mammalian MIPs newly identified in this study (from left-to-right: CFAP68, CFAP77, CFAP90, FAM166C, and FAM183A). (*E*) Model and density for Tektin 1-4. (*F*) FSC curves for the consensus reconstruction of the 8-nm repeat of human sperm flagella singlet microtubules (SMT consensus, black), and the best resolved reconstruction after applying a two-protofilament mask (SMT local, red). (*G*) Map of the sperm SMT consensus refinement colored by local resolution. The circular inset shows the improvement in local resolution following focused refinement. **H**. Model and density for SPACA9 (left) and Helix H2 (right) in the human sperm SMT. In panels A and F, the resolution was determined by the FSC=0.143 criterion (1). Landmark residues are labeled in panels (C-E) and (H).

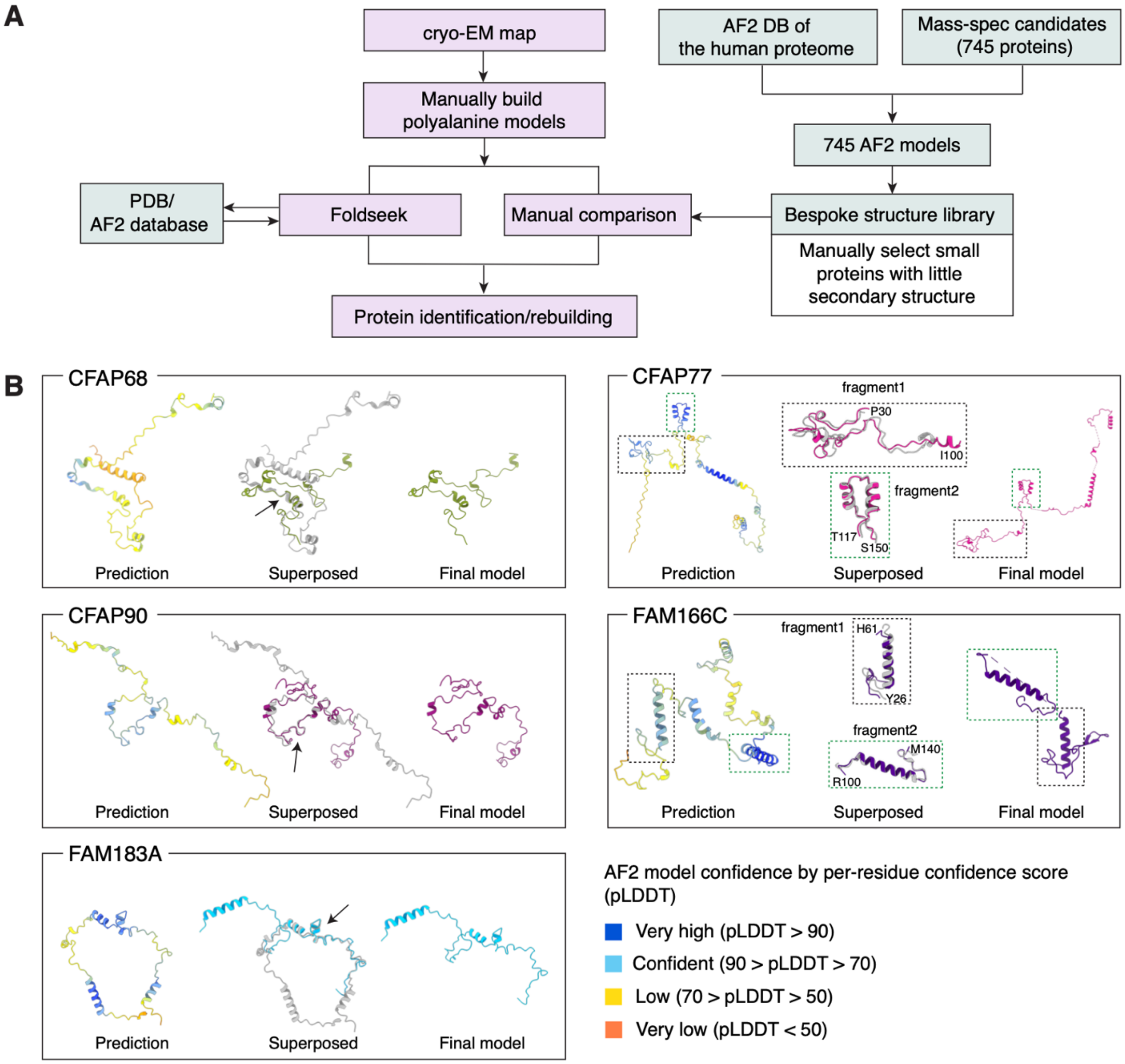

Fig. S3. Use of AlphaFold2 (AF2) in the modeling process. (*A*) A flow diagram showing how structural predictions from AF2 were used to identify and build atomic models of the microtubule inner proteins (MIPs). (*B*) Comparison on the AF2 predictions with the final atomic models for CFAP68, CFAP77, CFAP90, FAM166C and FAM183A. The residues of the AF2 predictions are colored by per-residue confidence score (pLDDT). The central panel shows an overlay between the prediction and final model. Similar regions are either indicated with an arrow or boxed. These superpositions demonstrate that although AF2 predictions of MIPs are imperfect, they do predict features, such as the length of α-helices, that can be used to help identify proteins in cryo-EM maps.

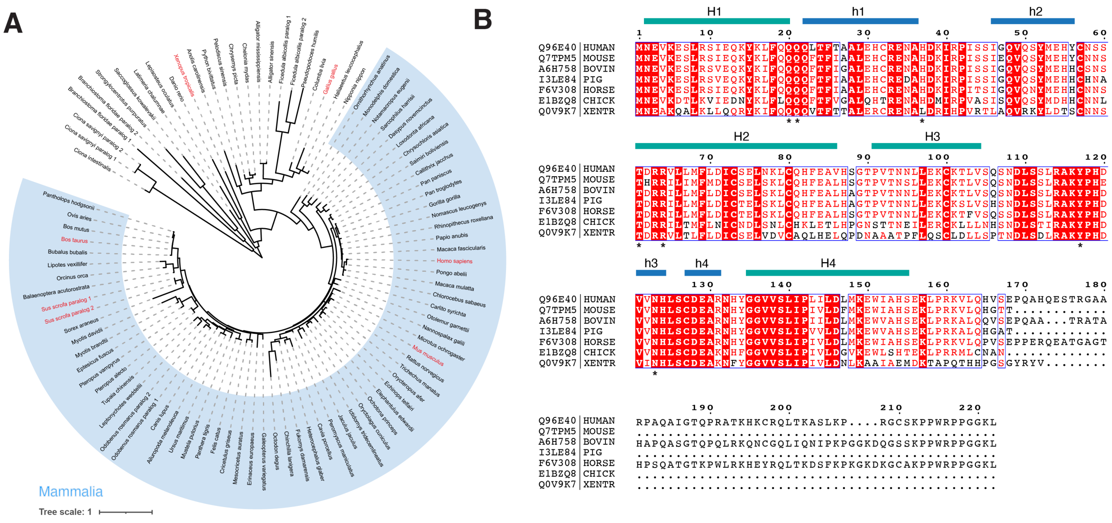

Fig. S4. SPACA9 sequence conservation. (*A*) Phylogenetic tree of SPACA9. The phylogenetic tree was extracted from EggNOG 5.0.0 (accession number: ENOG502QQV9) (2) and visualized by iTOL v.6.5.2 (3). Some species including pigs have two SPACA9 paralogs. Mammalian species are shaded blue. Names of species analyzed by cryo-EM/ET in this paper are highlighted red. (*B*) Sequence alignment performed using Clustal Omega v.1.2.2 (4) and visualized in ESPrint v.3.0 (5). One of the two pig SPACA9 paralogs was used for sequence alignment. Helices are annotated above the aligned sequences according to the tertiary structure of human SPACA9. Conserved residues that interact with tubulin and SAXO protein(s) are marked with asterisks.

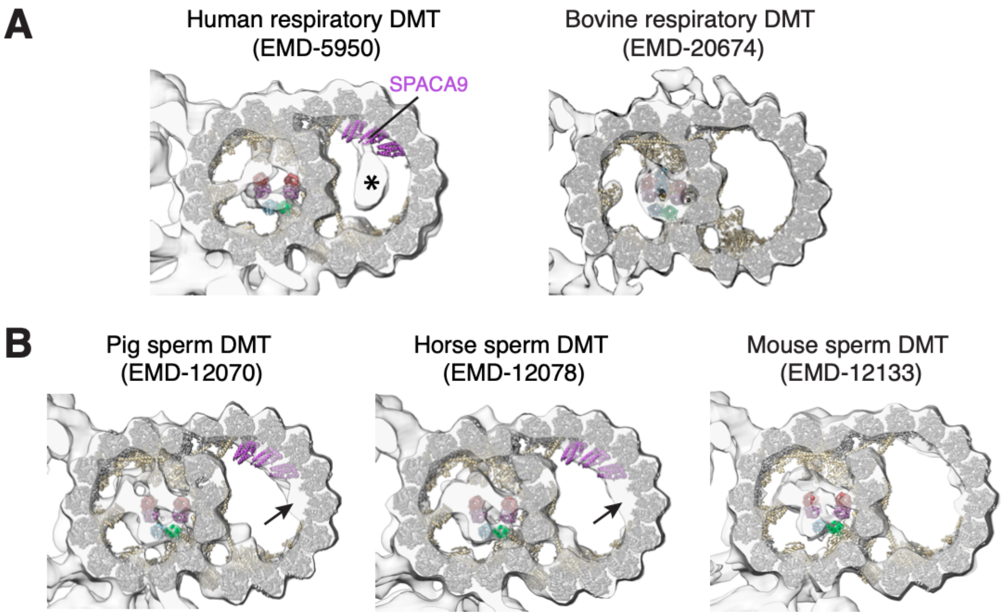

Fig. S5. Analysis of subtomogram averages of mammalian doublet microtubules (DMTs). (*A*) Transparent isosurface renderings of subtomogram averages of human (left) and bovine (right) respiratory DMTs with their cryo-EM-derived atomic models fitted inside. SPACA9 was not observed in the bovine respiratory DMT. Interestingly, the human model is not a perfect fit to the human DMT subtomogram average with differences at the tektin bundle and SPACA9. There is an intralumenal density in the subtomogram average (marked with an asterisk) that was not observed by single-particle cryo-EM. (*B*) Subtomogram averages of pig (left), horse (middle) and mouse (right) sperm DMTs with the atomic model of the human respiratory DMT fitted inside. Pig and horse sperm DMTs have density that could accommodate an additional SPACA9 subunit, indicated with an arrow. Density for SPACA9 is not observed in the B tubule of the mouse sperm DMT.

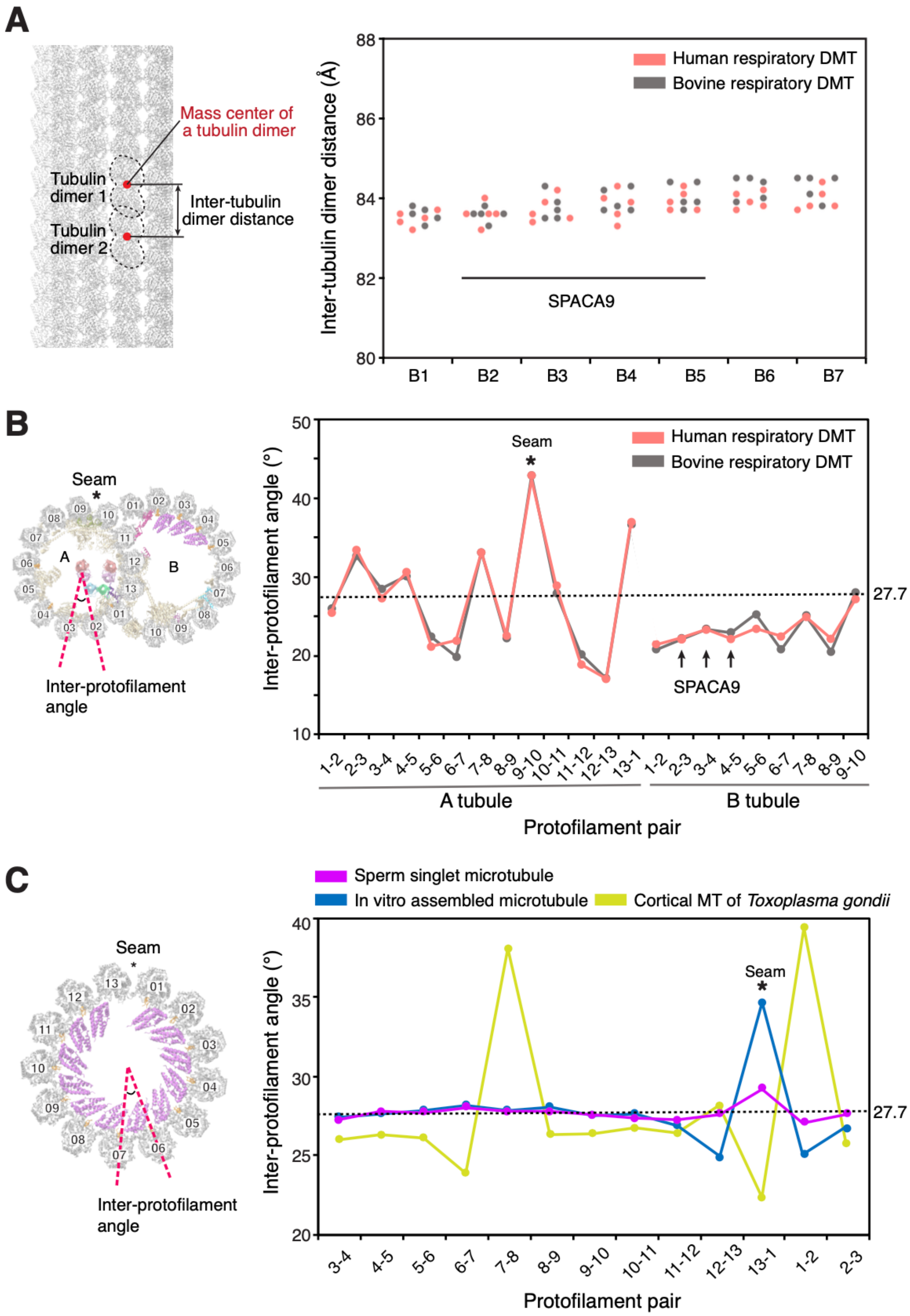

Fig. S6. Analysis of microtubule geometry. (*A*) Plot of inter-tubulin dimer distances for B-tubule protofilaments from the human and bovine DMT. Each protofilament has five datapoints, corresponding to the measurements between distinct tubulin heterodimer pairs in a single 48-nm repeat. Protofilaments B2-B5 are bound by SPACA9 in the human DMT but are undecorated in the bovine DMT. As shown by the schematic on the left, distances were calculated between mass

centers of the tubulin heterodimers. (*B*) Plot of the inter-protofilament angles calculated from the atomic models of the human and bovine respiratory DMT. (*C*) Plot of the inter-protofilament angles of human sperm SMT compared with in vitro assembled GDP microtubules (6) and the cortical microtubules of *T. gondii* (7). In panels (B) and (C), the inter-protofilament angle is represented by the relative rotation of tubulin heterodimers of adjacent protofilaments and was calculated using the *angle_between_domains* command in PyMOL. The dashed black line at 27.7° indicates the average inter-protofilament angles for a symmetric 13-protofilament tubule (360° divided by 13).

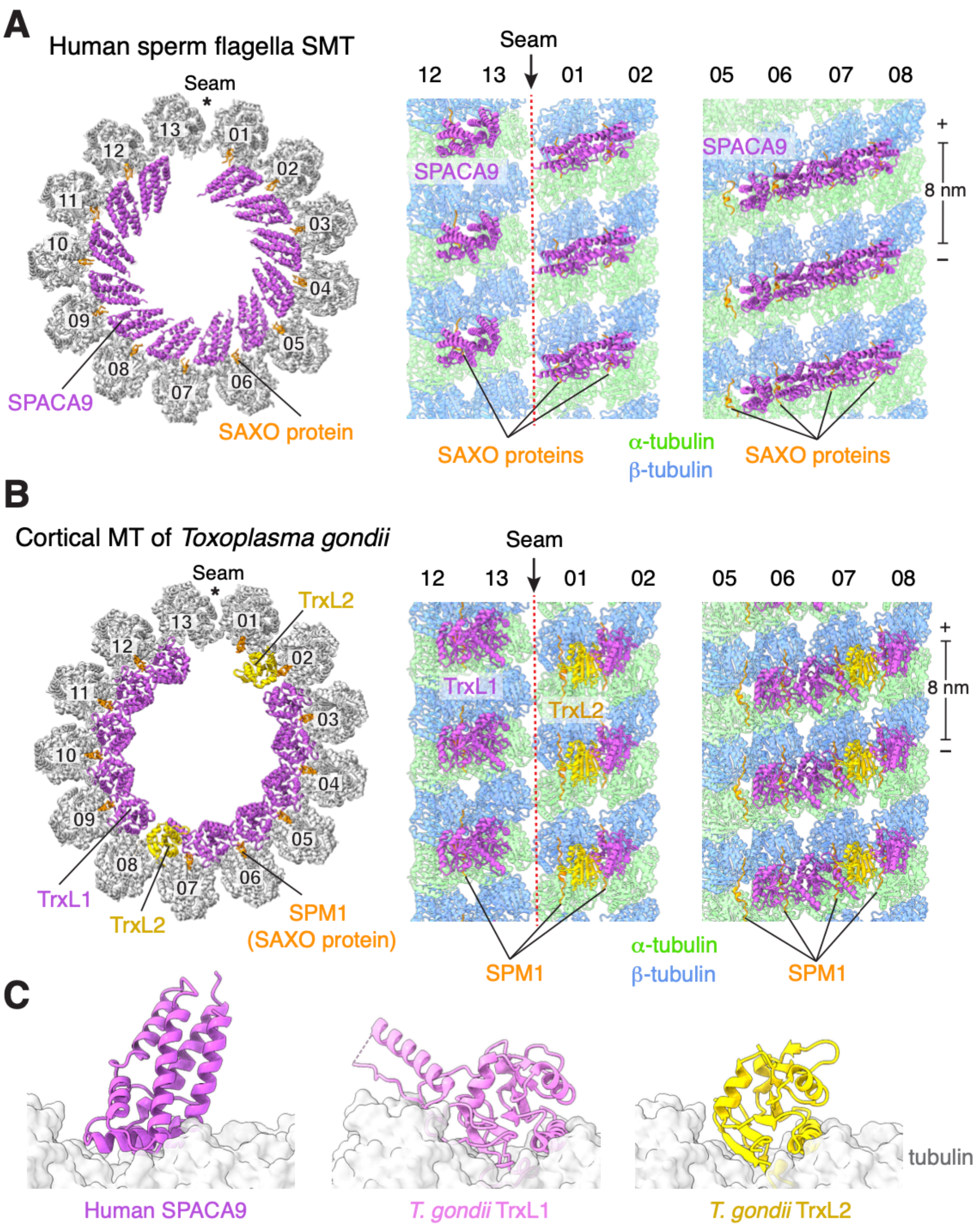

Fig. S7. Comparison of TAILS with the microtubule inner proteins (MIPs) of the cortical microtubules of *Toxoplasma gondii*. (*A*) Cross-section (left) and longitudinal views (right) of the atomic model of the SMT from the distal region of human sperm flagella showing the arrangement of TAILS formed by 12 copies of SPACA9 and SAXO proteins. SPACA9 binds to all protofilament pairs except those at the microtubule seam. (*B*) Cross-section (left) and longitudinal views (right) of the atomic model of the cortical microtubule of *Toxoplasma gondii* (PDB: 7MIZ) (7) showing a TAILS-like structure formed by 10 copies of TrxL1, 2 copies of Trxl2 and the SAXO protein, SPM1. TrxL1 and TrxL2 binds to all protofilament pairs except those at the microtubule seam. (*C*) The tertiary structure of SPACA9 (left) bears no resemblance to TrxL1 (middle) or TrxL2 (right) despite engaging similar lumenal surfaces of the microtubule.

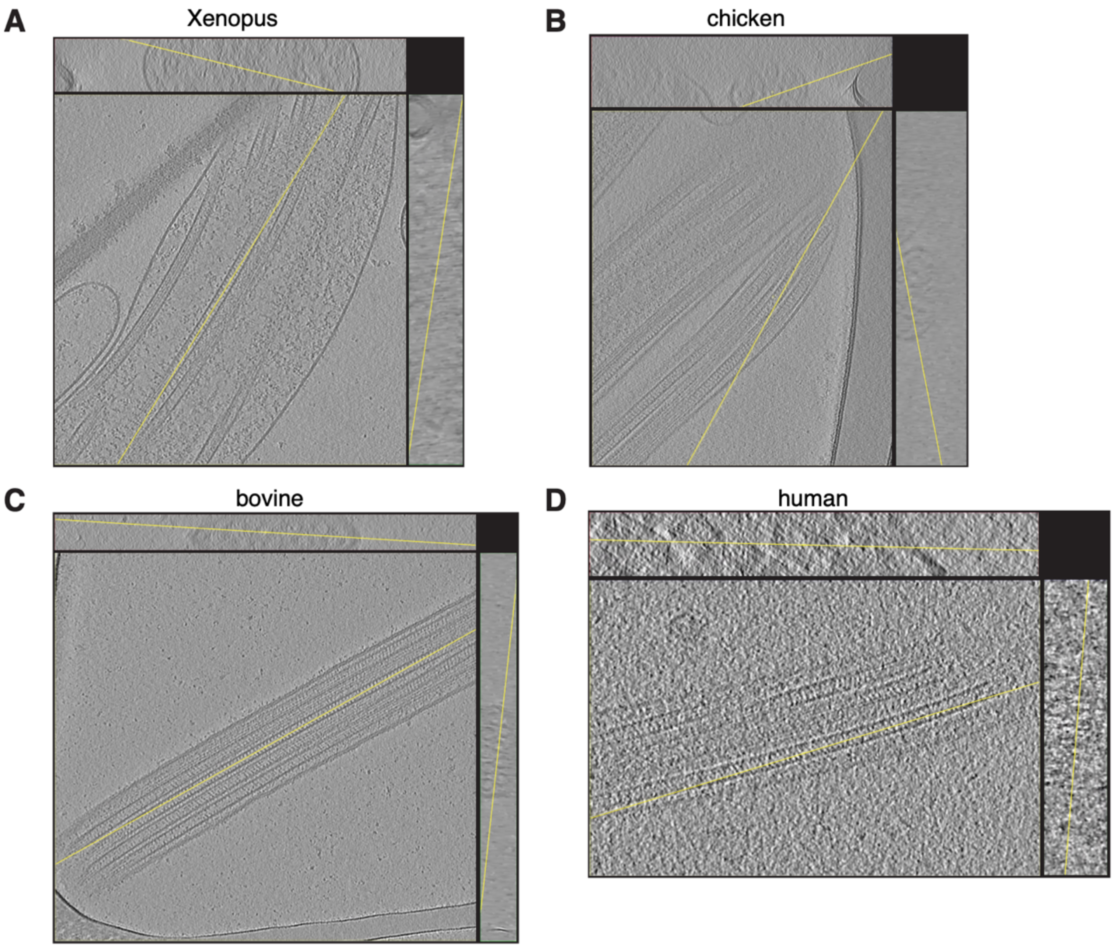

Fig. S8. Tomograms of animal sperm. XYZ views of (*A*) frog sperm, (*B*) chicken sperm, (*C*) bull sperm, and (*D*) human sperm. Sections of these tomograms are shown in Fig. 6B.

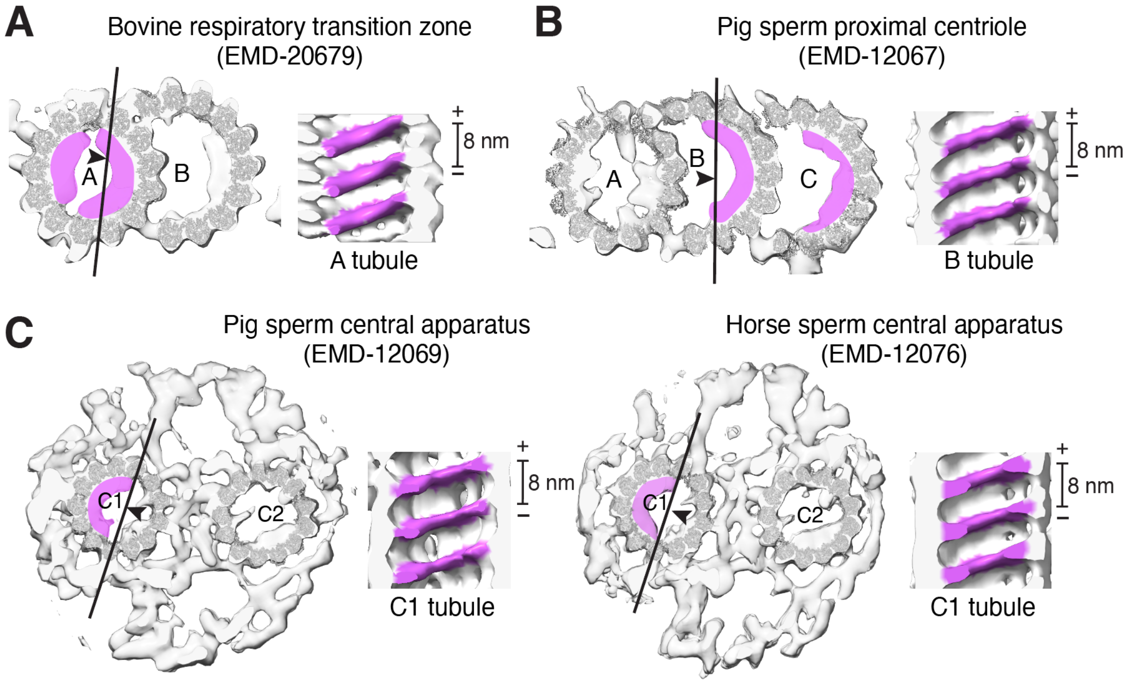

Fig. S9. 8-nm repeating striations are found in other microtubules in the centrosome-cilium complex. (*A*) Isosurface rendering of the cross section of a subtomogram average of a doublet microtubule (DMT) from the bovine respiratory cilia transition zone (EMD-20679) (8). Striations with 8-nm periodicity in the A tubule and are shown in the longitudinal slice and colored purple. (*B*) Cross section and longitudinal slice of the subtomogram average of a triplet microtubule (TMT) of the pig sperm proximal centriole (EMD-12067) (9). Striations with 8-nm periodicity are found in the B and C tubules. (*C*) Cross sections of subtomogram averages of pig (EMD-12069, left) and horse (EMD-12076, right) central apparatuses (CA) (9). Striations with 8-nm periodicity are found in the C1 microtubule (shown in the longitudinal slices).

SI Tables

Table S1. Statistics for data collection, data processing, model refinement and validation.

	**48-nm repeat of human respiratory DMT**	**8-nm repeat of human sperm SMT**	**Tomography of animal sperm**
**Data collection**			
Facility	Harvard Cryo-EM Center for Structural Biology	Arnold and Mabel Beckman Cryo-EM Center, University of Washington	Umeå Core Facility Electron Microscopy, Sweden
Microscope	Titan Krios	Titan Krios	Titan Krios
Detector	K3	K3	K2
Voltage (keV)	300	300	300
Nominal magnification	64,000×	105,000×	33,000×
Detector mode	Counting	Super-resolution	Counting
Electron exposure ($e^-/Å^2$)	60	40	100 (81 for chicken sperm) over 61 tilt images.
Defocus range set during data acquisition (μm)	-0.8 to -2	-1 to -3	-4 to -6
Pixel size (Å)	1.37	0.4215	4.37
**Data Processing**			
Movie stacks	37,071	1,136	
Final particles	208,558	21,990	
Map resolution of consensus refinement (Å)	3.6	6.0	
Map resolution range of local masked refinements (Å)	3.1-4.0	4.5-6.5	
**Model composition**			
Chains	435	109	
Atoms	1,265,783	290,845	
Residues	160,638	37,576	
Ligands	149 GTP/ 153 GDP/ 149 Mg	38 GTP/ 38 GDP/ 38 Mg	
**Refinement**			
Resolution limit set in refinement (Å)	3.6	6	
Correlation coefficient (CCmask)	0.74	0.57	
C_{ref} (masked) (Å)	3.8	6.4	
Root-mean-square deviation (bond lengths) (Å)	0.005	0.005	
Root-mean-square deviation (bond angles) (Å)	1.135	1.166	
**Validation**			
MolProbity Score	1.48	1.72	
Clashscore	4.93	7.8	
Rotamer outliers (%)	0.13	0.17	
Ramachandran (favored) (%)	96.54	95.81	
Ramachandran (outliers) (%)	0.07	0.07	

Table S2. Microtubule inner proteins (MIPs) of human respiratory doublet microtubules (DMTs) identified using cryo-EM.

Human MIPs	UniProt ID	Alias	*Chlamydomonas* ortholog	Molecular mass in Da / (number of residues)	Periodicity	Protofilament location	Built residues#
CFAP20	Q9Y6A4	C16orf80	FAP20	22,774 (193)	8 nm	Inner junction	1-186
CFAP21	Q8N7U6	EFHB	FAP21	93,802 (833)	48 nm	A08-A10	240-641, 665-710, 717-822
CFAP45	Q9UL16	CCDC19	FAP45	65,730 (551)	48 nm	B07-B10	76-94, 105-543
CFAP52	Q8N1V2	WDR16	FAP52	68,298 (620)	16 nm	B09-B10	11-620
CFAP53	Q96M91	CCDC11	FAP53	61,835 (514)	48 nm	A06-A11	43-505
CFAP68*	Q9H5F2	C11orf1	FAP68	17,785 (150)	48 nm	A09-A10	60-148
CFAP77*	Q6ZQR2-2†	C9orf171	FAP77	32,979 (284)	16 nm	B01-B02, outside A11-A12	24-152, 159-210, 217-227, 236-269
CFAP90*	A4QMS7	C5orf49	FAP90	16,991 (147)	48 nm	B09-B10	20-146
CFAP95	Q5VTT2	C9orf135	FAP95	26,445 (229)	48 nm	A09-A11	30-59, 77-124, 152-167, 176-228
CFAP107	Q8N1D5	C1orf158	FAP107	23,033 (194)	48 nm	A09-A11	8-175
CFAP126	Q5VTH2	C1orf192, FLTP	FAP126	19,293 (177)	16 nm	Outside A12-A13	1-117
CFAP141	Q5VU69	C1orf189	FAP141	12,131 (101)	48 nm	A09-A10	52-101
CFAP161	Q6P656	C15orf26	FAP161	34,294 (301)	2 copies every 48 nm	A09-A11	3-279
CFAP210	Q0VFZ6	CCDC173	FAP210	66,403 (552)	48 nm	B06-B10	46-551
CFAP276	Q5T5A4	C1orf194	FAP276	19,350 (169)	16 nm	B09-10, outside A13-A01	33-101, 127-169
EFHC1	Q5JVL4	-	RIB72	73,990 (640)	16 nm	A12-A05	9-51, 85-369, 379-535
EFHC2	Q5JST6	-	RIB72	87,397 (749)	16 nm	A12-A05	3-267, 274-379, 389-425, 433-610, 617-704, 721-748
ENKUR	Q8TC29	CFAP106	FAP106	29,454 (256)	16 nm	B09-B10, outside A12-A13	4-253
FAM166B	A8MTA8	-	-	30,575 (275)	8 nm	A01-A05	15-63, 88-118, 166-211, 253-272
FAM166C*	A6NJV1	C2orf70	-	23,421 (201)	16 nm	A13-A01	2-61, 100-140
FAM183A*	A6NL82	-	FAP144	15,996 (134)	48 nm	B07-B08	10-120
MNS1	Q8NEH6	SPATA40	FAP127	60,571 (495)	48 nm	A07-A11	6-485
NME7	Q9Y5B8	CFAP67, NM23-H7	FAP67	42,492 (376)	2 copies every 48 nm	A09	5-375
PACRG	Q96M98-2†	-	PACRG	29,312 (257)	8 nm	Inner junction	38-257
Pierce1	Q5BN46	C9orf116	FAP182	15,260 (136)	48 nm	A07-A08	24-136
Pierce2	H3BRN8	C15orf65	FAP182	13,763 (121)	48 nm	A07-A08	26-119
RIBC2	Q9H4K1	-	RIB43a	45,427 (382)	2 copies every 48 nm	A11-A13	15-382
SPACA9*	Q96E40	C9orf9	-	25,166 (222)	3 copies every 8 nm	B02-B05	1-160
SPAG8	Q99932	SPAG3	FAP143	51,139 (485)	48 nm	A09-A13	276-454
Tektin 1	Q969V4	-	-	48,283 (418)	16 nm	A tubule lumen	6-399
Tektin 2	Q9UIF3	TEKTB1	-	49,672 (430)	16 nm	A tubule lumen	8-399
Tektin 3	Q9BXF9	-	-	56,636 (490)	16 nm	A tubule lumen	92-486
Tektin 4	Q8WW24	-	-	50,649 (435)	16 nm	A tubule lumen	38-435

*MIPs newly identified in this study.
# Underscored residues have side chains truncated due to the map quality. Side chains of SPACA9 between protofilaments B04-B05 and the innermost tektin 2:tektin 4 dimer are also truncated.
†Isoform sequences of CFAP77 and PACRG were assigned based on side chain density. Canonical sequences of other proteins were used for modeling.

Dataset S1. Mass spectrometry data. List of proteins identified by mass spectrometry in the human respiratory axoneme sample. Proteins unambiguously identified in the cryo-EM map are highlighted in yellow. CFAP141, FAM166C, Pierce1 and SPAG8 were identified in the cryo-EM map but were not detected in the mass spectrometry analysis potentially due to their small size or low abundance.

Dataset S2. Measurements of SPACA9 tilt angle, inter-protofilament angle and inter-tubulin dimer distance.

SI References

1. P. B. Rosenthal, R. Henderson, Optimal determination of particle orientation, absolute hand, and contrast loss in single-particle electron cryomicroscopy. *Journal of Molecular Biology* 333, 721–745 (2003).

2. J. Huerta-Cepas, *et al.*, eggNOG 5.0: a hierarchical, functionally and phylogenetically annotated orthology resource based on 5090 organisms and 2502 viruses. *Nucleic Acids Research* 47, D309–D314 (2019).

3. I. Letunic, P. Bork, Interactive Tree Of Life (iTOL) v5: an online tool for phylogenetic tree display and annotation. *Nucleic Acids Research* 49, W293–W296 (2021).

4. F. Sievers, *et al.*, Fast, scalable generation of high-quality protein multiple sequence alignments using Clustal Omega. *Molecular systems biology* 7, 539 (2011).

5. X. Robert, P. Gouet, Deciphering key features in protein structures with the new ENDscript server. *Nucleic Acids Research* 42, W320-4 (2014).

6. R. Zhang, B. LaFrance, E. Nogales, Separating the effects of nucleotide and EB binding on microtubule structure. *Proc. Natl. Acad. Sci. U.S.A.* 115, E6191–E6200 (2018).

7. X. Wang, *et al.*, Cryo-EM structure of cortical microtubules from human parasite Toxoplasma gondii identifies their microtubule inner proteins. *Nat Commun* 12, 3065–14 (2021).

8. G. A. Greenan, R. D. Vale, D. A. Agard, Electron cryotomography of intact motile cilia defines the basal body to axoneme transition. *J. Cell Biol.* 219, 241 (2020).

9. M. R. Leung, *et al.*, The multi-scale architecture of mammalian sperm flagella and implications for ciliary motility. *EMBO J.* 40, e107410 (2021).
